# Molecular characterization of hematopoietic stem cells after in vitro amplification on biomimetic 3D PDMS cell culture scaffolds

**DOI:** 10.1038/s41598-021-00619-6

**Published:** 2021-10-27

**Authors:** Lisa Marx-Blümel, Christian Marx, Jürgen Sonnemann, Frank Weise, Jörg Hampl, Jessica Frey, Linda Rothenburger, Emilio Cirri, Norman Rahnis, Philipp Koch, Marco Groth, Andreas Schober, Zhao-Qi Wang, James F. Beck

**Affiliations:** 1 Department of Pediatric Hematology and Oncology, Children’s Clinic, Jena University Hospital, Am Klinikum 1, 07747 Jena, Germany; 2 Research Center Lobeda, Jena University Hospital, Jena, Germany; 3 Leibniz Institute on Aging-Fritz Lipmann Institute (FLI), Jena, Germany; 4 Institute for Micro and Nanotechnologies MacroNano®, Nano-Biosystem Technology, Ilmenau University of Technology, Ilmenau, Germany; 5 Faculty of Biological Sciences, Friedrich-Schiller-University of Jena, Jena, Germany

**Keywords:** Proteomics, Cell signalling, Structural properties, Haematopoietic stem cells, Transcriptomics

## Abstract

Hematopoietic stem cell (HSC) transplantation is successfully applied since the late 1950s. However, its efficacy can be impaired by insufficient numbers of donor HSCs. A promising strategy to overcome this hurdle is the use of an advanced ex vivo culture system that supports the proliferation and, at the same time, maintains the pluripotency of HSCs. Therefore, we have developed artificial 3D bone marrow-like scaffolds made of polydimethylsiloxane (PDMS) that model the natural HSC niche in vitro. These 3D PDMS scaffolds in combination with an optimized HSC culture medium allow the amplification of high numbers of undifferentiated HSCs. After 14 days in vitro cell culture, we performed transcriptome and proteome analysis. Ingenuity pathway analysis indicated that the 3D PDMS cell culture scaffolds altered PI3K/AKT/mTOR pathways and activated SREBP, HIF1α and FOXO signaling, leading to metabolic adaptations, as judged by ELISA, Western blot and metabolic flux analysis. These molecular signaling pathways can promote the expansion of HSCs and are involved in the maintenance of their pluripotency. Thus, we have shown that the 3D PDMS scaffolds activate key molecular signaling pathways to amplify the numbers of undifferentiated HSCs ex vivo effectively.

## Introduction

Human hematopoietic stem cells (HSCs) are able to produce all types of blood cells and are defined by two key properties: their ability of self-renewal and potential of lineage specific differentiation^1,2^. Hence, HSCs from the bone marrow (BM), peripheral blood (PB) or umbilical cord blood (UCB) are an appealing source for stem cell-based therapies such as HSC transplantation (HSCT)^3,4^. Since its first application in the 1950s, HSCT has become a potentially life-saving treatment option for patients with otherwise incurable diseases like chemoresistant high-risk malignancies and a broad variety of non-malignant congenital and acquired disorders, such as BM failures, hemoglobinopathies, primary immune deficiencies or autoimmune diseases^4,5^. Huge efforts have been made to improve the efficacy of HSCT. A promising approach relies on the transplantation of high numbers of pluripotent stem cells^6^, which can be achieved by an ex vivo HSC expansion before transplantation. However, the attempts to expand pluripotent HSCs in vitro were of limited success so far^3,4^, because HSCs rapidly differentiate ex vivo to become committed progenitor cells.

Several approaches were done to improve HSC culture conditions using optimized culture media. These advanced cultivation protocols include the use of hematopoietic cytokines, developmental regulators and/or chemical compounds to maintain HSC pluripotency. However, this was not sufficient to support both, the ability of self-renewal and effective amplification of HSCs in vitro^3,7–10^. On the other hand, some approaches rely on the use of 3D cultivation substrates that mimic the natural BM niche, based on the hypothesis that the culture microenvironment may play a critical role in the expansion of HSCs in vitro^7,11–14^. Various natural biomaterials and synthetic scaffolds have been described to create close-to-nature microenvironments for an improved control of stem cell response in vitro^7,14–16^.

The activity of molecular signaling pathways safeguards the HSC maintenance and their self-renewal. One of the most studied pathways is the PI3K/AKT/mTOR signaling. A balanced activation of this pathway promotes HSC functions and maintenance. However, defects or hyper-activation of PI3K/AKT/mTOR signaling can lead to severe HSC dysfunctions and differentiation^17–20^. The forkhead O (FOXO) family of transcription factors and hypoxia-inducible factor 1-alpha (HIF1α) as part of the PI3K/AKT/mTOR signaling pathway control cell cycle progression, autophagy, cell metabolism and the cellular redox state thereby positively regulating the self-renewal of HSCs^21–26^. Recent studies show that the metabolism of fatty acids (FAs) and particularly of cholesterol, which is regulated by PI3K/AKT/mTOR and connected sterol regulatory element-binding protein (SREBP) and HIF1α signaling pathways^27^, is important to expand high numbers of undifferentiated HSCs ex vivo^8^.

We recently established a novel 3D polydimethylsiloxane (PDMS) ex vivo cell culture system to obtain high numbers of human HSCs and in parallel to maintain their pluripotency^7^. Briefly, we optimized the HSC medium by supplementation with a panel of hematopoietic cytokines and valproic acid (VPA), a histone deacetylase (HDAC) inhibitor, which separately promote stem cell maintenance in vitro^3,28^. In addition, we developed 3D PDMS scaffolds based on the BM structure of a human long bone cross section and, hence, mimicking the natural stem cell niche^7^.

In the present study, we investigated specific molecular signaling pathways that contribute to the HSC maintenance and amplification on 3D PDMS cell culture scaffolds. To this end, we compared HSCs after 14 days in vitro culture on 3D PDMS with cells grown on 2D polystyrene (PS), which is the commonly used material for HSC ex vivo culture, or on silicon oxide-coated 3D PDMS (3D SiOn), and to the initial HSC populations before cell culture. Here we show that non-coated 3D PDMS scaffolds activate SREBP, HIF1α and FOXO signaling pathways in cells during ex vivo expansion and enable to maintain high numbers of naïve long term (LT)-HSCs.

## Results

### 3D PDMS scaffolds offer the best conditions for ex vivo HSC expansion

**Figure 1:**
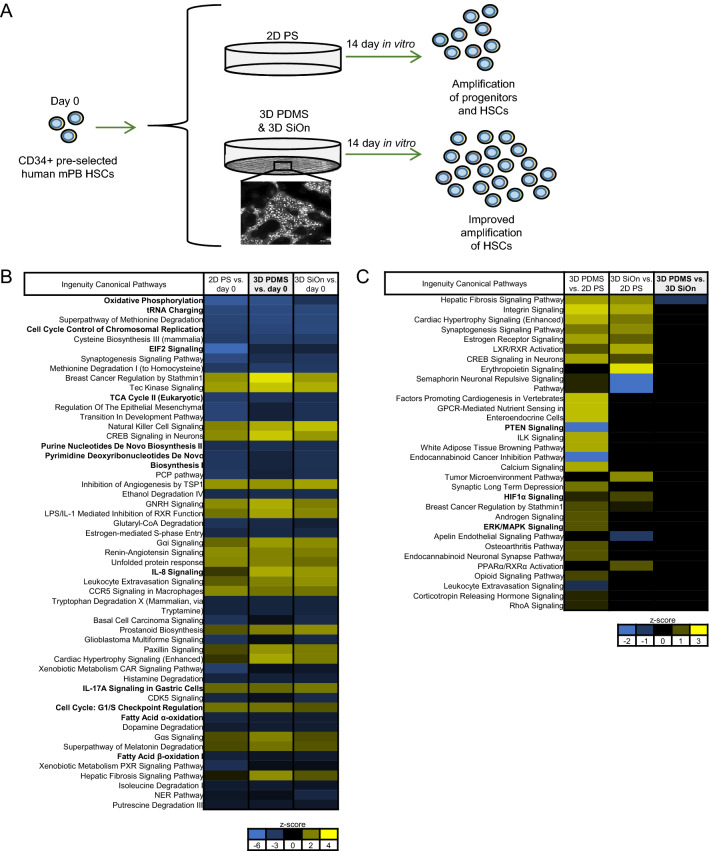
(**A**) Graphical summary of the in vitro cell culture system used in this study. CD34+ pre-selected human hematopoietic stem cells from mobilized peripheral blood (mPB HSCs) were cultivated for 14 days in vitro within an optimized HSC culture medium [supplemented with a panel of cytokines and valproic acid (VPA)]. HSCs were grown either on standard 2D polystyrene (PS), or on non-coated as well as silicon oxide (SiOn)-coated 3D polydimethylsiloxane (PDMS) cell culture scaffolds mimicking the natural bone marrow (BM) structure and stem cell niche to expand high amounts of undifferentiated HSCs during ex vivo culture. (**B**–**C**) The transcriptome of the initial HSC population at day 0 and after 14 days in vitro on 2D PS, 3D PDMS and 3D SiOn scaffolds was analyzed by RNA sequencing (with 3 replicates per group), and the data sets were post-analyzed using ingenuity pathway analysis (IPA). (**B**) Activation of cellular pathways (top 50) after 14 days in vitro on 2D PS, 3D PDMS and 3D SiOn scaffolds compared to day 0 (analysis cutoff: *q* < 0.05, log2-fold change > 1) and (**C**) activation of cellular pathways (top 30) after 14 days in vitro in comparison between 2D PS, 3D PDMS and 3D SiOn scaffolds (analysis cutoff: *q* < 0.05) is indicated by corresponding z-scores (blue inactivation/yellow activation) and ranked by the − log(p-value) of individual pathways (most significant at the top). Important pathways/molecules are highlighted with bold text.

In our previous study^7^, we showed that our novel 3D PDMS structures offered superior in vitro HSC culture conditions and expanded the numbers of CD34+ cells compared with 2D PS as well as unstructured 2D PDMS significantly (Fig. 1A). Moreover, we showed that particularly non-coated 3D PDMS scaffolds increased the numbers of naïve LT-HSCs, which was also reflected in colony forming units (CFU) assays^7^.

In our current study, we analyzed the effect of the scaffold material and cell culture environment on HSC expansion. PS is the established standard material for HSC in vitro culture, and it could offer beneficial properties in a 3D system compared to PDMS. We produced 3D PS cell culture scaffolds based on our 3D PDMS scaffolds and analyzed the numbers of CD34+ HSCs after 14 days in vitro by flow cytometry (Supplementary Fig. S1A). Since we used primary human HSCs from different individuals, the experimental data showed a certain variability. Confirming our previous results^7^, both 3D SiOn and 3D PDMS scaffolds maintained a higher percentage of CD34+ cells and increased the total number and amplification of CD34+ cells compared to 2D PS. Although CD34 is generally used as a marker to discriminate between stem cells and differentiated cells, it alone is insufficient to label naïve HSCs. Therefore, we included additional markers for HSC immaturity^29^. 3D PDMS gave rise to the best amplification of LT-HSCs, as shown by an increase of total cell number and percentage of CD34+/CD38−/CD45RA−/CD49f+/CD90+ LT-HSCs after 14 days in vitro (Supplementary Fig S1B). 3D PS showed similar effects on the expansion of CD34+ cells and LT-HSCs as 2D PS (Supplementary Fig. S1A,B). Due to the limited number of donors in this analysis, the observed effects were statistically not significant.

To assess the clonogenic potential of 3D PS, CFU assays were performed with cells after 14 days in vitro (Supplementary Fig. S1C). Cells from 2D PS, 3D PS and 3D PDMS all yielded higher total colony counts compared with 3D SiOn (Supplementary Fig. S1C). CFU-E colonies were only formed by cells from 2D PS and 3D SiOn. Cells grown on 3D PS and 3D PDMS showed more CFU-M and CFU-GM colonies than cells from 3D SiOn or 2D PS. The highest number of CFU-GM were observed in cells grown on 3D PDMS, and the highest number of CFU-G was found in cells from 2D PS. Nevertheless, we only found BFU-E colonies and higher numbers of CFU-GEMM colonies (*p* < 0.05 to 3D PS) in cells grown on 3D PDMS. These results are consistent with data obtained by flow cytometry (Supplementary Fig. S1B) and our previous work^7^ showing that cells grown on 3D PDMS have the highest number of immature LT-HSCs and the highest repopulation potential after 14 days in vitro. Consequently, we discontinued the use of 3D PS.

### Multi-omics analysis indicate the activation of specific molecular signaling pathways after 14 days in vitro

To understand the molecular basis of how 3D PDMS structures supported the expansion of naïve HSCs during 14 days in vitro, we performed transcriptome and proteome analysis of cells that were grown on 2D PS, 3D PMDS or 3D SiOn and of the initial CD34-enriched HSC population at day 0.

RNA sequencing revealed greater transcriptome differences in cells after 14 days in vitro and at day 0 than between the individual cell culture scaffolds (Fig. 1B,C, Supplementary Figs. S2, S3). This is congruent with the notion that HSCs differentiate during in vitro culture^7^, which is reflected by decreased percentages of CD34+ cells (Supplementary Fig. S1A). Principal component analysis (PCA) of the transcriptome data sets showed that HSCs at day 0 and after 14 days in vitro formed two distinct groups irrespective of the cell culture scaffold based on different gene expression patterns between the cells before and after ex vivo expansion (Supplementary Fig. S2A). Volcano plots of the individual comparisons between cells after 14 days in vitro and day 0 similarly showed highly altered transcripts for each cell culture surface (Supplementary Fig. S2B–D). Nevertheless, cells from both 3D scaffolds formed distinct groups compared with 2D PS after 14 days in vitro, but were nearly indistinguishable among each other (Supplementary Fig. S3).

Ingenuity pathway analysis (IPA) of cells after 14 days in vitro and day 0 showed that all cultivation scaffolds altered similar molecular signaling pathways (ingenuity canonical pathways) and molecules (upstream regulators) regulating cell proliferation/cell cycle regulation, cell metabolism and immune response, particularly interleukin 8 (IL8) signaling (Fig. 1B, Supplementary Fig. S4A). Cells grown on 3D PDMS showed the strongest alterations of molecular pathways and regulators. We additionally observed inactivation of MYC, NOTCH1 and altered mechanistic target of rapamycin (mTOR) activation for all cultivation substrates after 14 days in vitro compared with day 0 (Supplementary Fig. S4A). IPA detected only small differences of signaling pathways and upstream regulators between cell populations expanded on 3D PDMS and 3D SiOn, in contrast to differences between both 3D scaffolds and 2D PS (Fig. 1C, Supplementary Fig. S4B). These alterations were mainly connected to an increased immune response, PI3K/AKT and downstream HIF1α and SREBP signaling.

Next, we analyzed the cell proteome by untargeted mass spectrometry. Consistently, we observed greater differences between cells after 14 days in vitro and at day 0 than between the cell culture surfaces (Supplementary Fig. S5). PCA and volcano plots of the proteome data sets confirmed that HSCs at day 0 and after 14 days in vitro formed two distinct groups irrespective of the cell culture scaffold (Supplementary Fig. S5A–D), and that both 3D scaffolds formed distinct groups compared with 2D PS, but were among each other nearly identical (Supplementary Fig. S5E–H).

**Figure 2:**
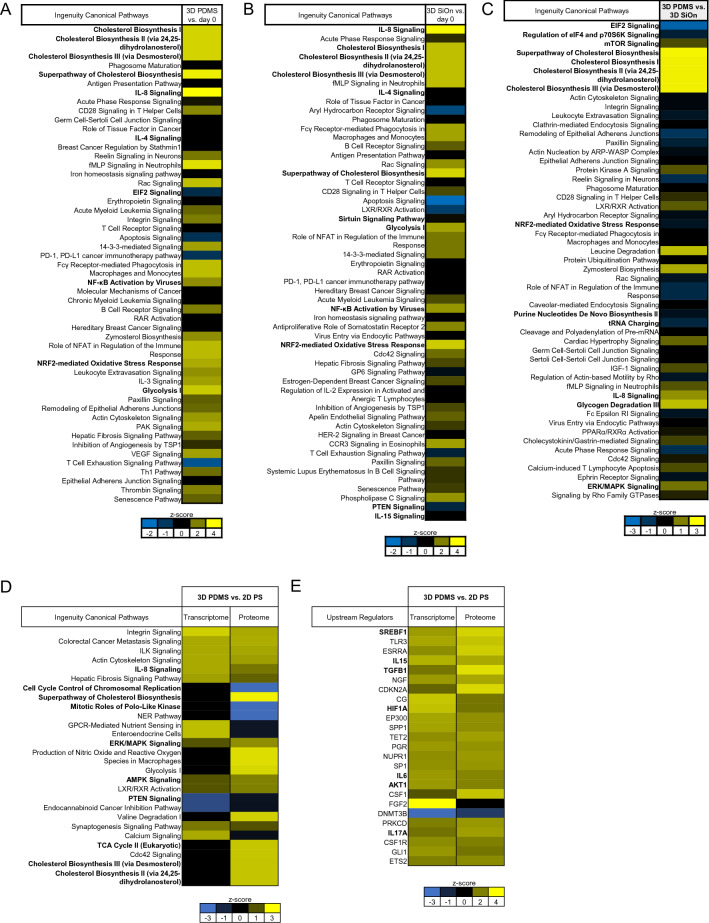
Human HSCs were cultivated for 14 days in vitro on 2D PS, 3D PDMS and 3D SiOn to expand high numbers of undifferentiated HSCs ex vivo. The proteome of the initial HSC population at day 0 and after 14 days in vitro on 2D PS, 3D PDMS and 3D SiOn scaffolds was analyzed by untargeted mass spectrometry (with 4 replicates per group) and the data sets were post-analyzed using IPA. Activation of cellular pathways (top 50) after 14 days in vitro on 3D PDMS (**A**) and 3D SiOn (**B**) scaffolds compared to day 0 (analysis cutoff: *q* < 0.05, log2-fold change > 0.5), and (**C**) activation of cellular pathways (top 50) after 14 days in vitro in comparison of 3D PDMS vs. 3D SiOn scaffolds (analysis cutoff: *q* < 0.05) is indicated by corresponding z-scores (blue inactivation/yellow activation) and ranked by the − log(p-value) of individual pathways (most significant at the top). (**D**,**E**) Transcriptome and proteome data sets were compared using IPA (analysis cutoff: *q* < 0.05). (**D**) Activation of similar cellular pathways (top 25) and (**E**) activation of similar regulatory molecules (upstream regulators; top 25) after 14 days in vitro in comparison of 3D PDMS vs. 2D PS scaffolds is indicated by corresponding z-scores and ranked by the − log(p-value) of individual molecules. Important pathways/molecules are highlighted with bold text.

IPA of cells after 14 days in vitro and day 0 showed that all cell culture scaffolds altered similar molecular signaling pathways involved in cell metabolism, particularly cholesterol metabolism, oxidative stress response and immune response/IL8 signaling (Fig. 2A,B, Supplementary Fig. S6A). Cells expanded on 3D PDMS showed the strongest activation of cholesterol metabolism. Alterations of cell proliferation/cell cycle regulation pathways were less abundant. The direct comparison between the cell culture surfaces after 14 days in vitro revealed that 3D PDMS specially induced the activation of FA/cholesterol metabolism and IL8 signaling, whereas cell grown on 3D SiOn had a stronger activation of EIF2 and mTOR signaling (Fig. 2C, Supplementary Fig. S6B,C).

Related to this, we observed a prominent activation of SREBP1/2 between HSCs at day 0 and after 14 days in vitro on all cell culture scaffolds (Supplementary Fig. S7). Cells grown on 3D PDMS showed the strongest activation of SREBP1/2 and connected regulatory molecules as well as of HIF1α. Moreover, we found downregulation of MYC, FOXO1 and NOTCH1 signaling and an altered mTOR activation after 14 days in vitro on all cell culture scaffolds by IPA (Supplementary Fig. S7). Of note, we also observed on all scaffolds an activation of CD38 after 14 days in vitro compared to day 0 (Supplementary Fig. S7A), reflecting the differentiation of HSCs during the in vitro expansion^29^.

A direct comparison of the transcriptome and proteome data sets using IPA showed that cells grown on 3D PDMS consistently had strongest activation of cholesterol metabolism, IL8, SREBP1/2, HIF1α and PI3K/AKT signaling (Fig. 2D,E, Supplementary Fig. S8A–D). In contrast, cells grown on 3D SiOn showed strongest immune response (Supplementary Fig. S8A,B).

### ELISA confirms a pro-inflammatory response in cell grown on 3D SiOn

**Figure 3:**
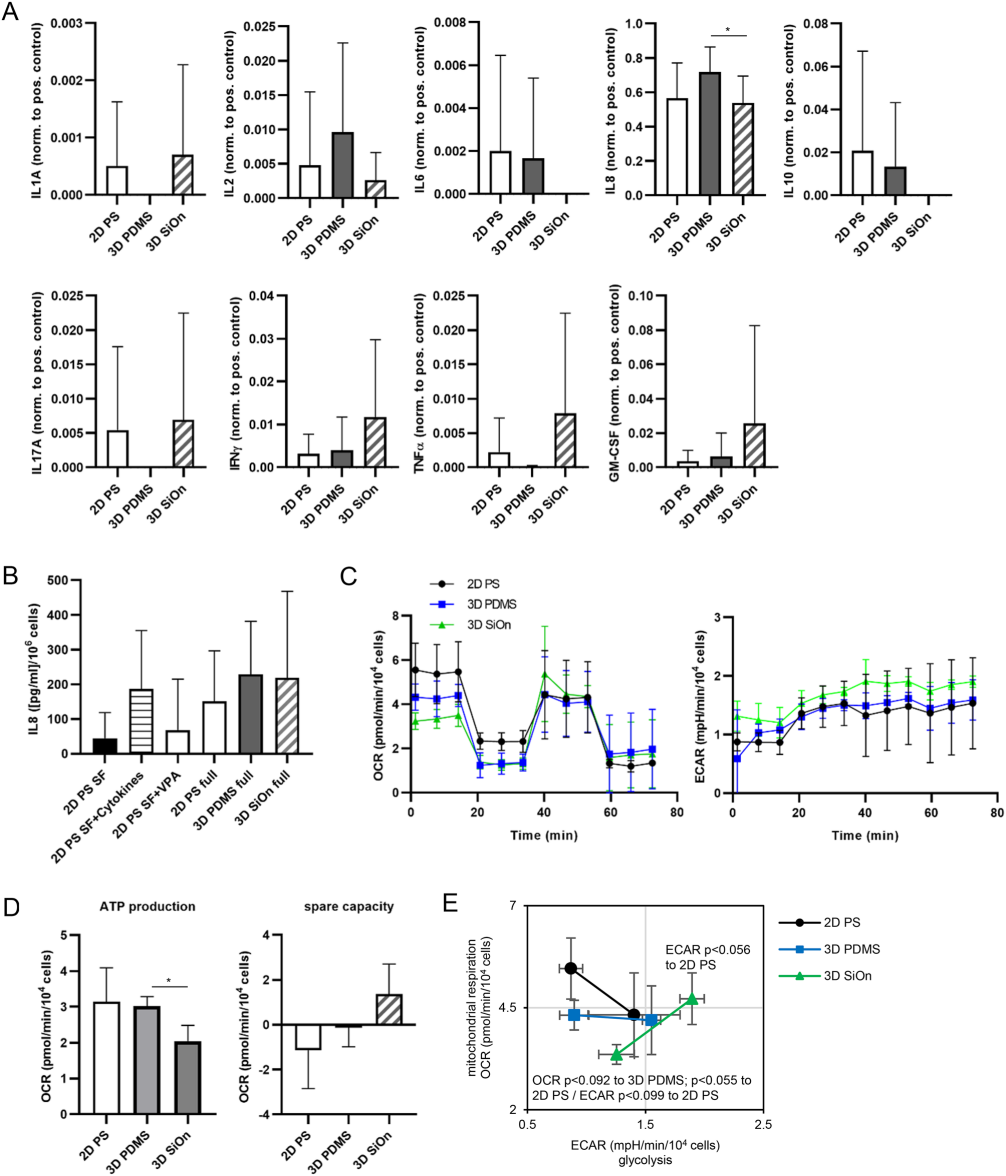
Human HSCs were cultivated for 14 days in vitro on 2D PS, 3D PDMS and 3D SiOn to expand high numbers of undifferentiated HSCs ex vivo. (**A**) After 14 days in vitro, a panel of indicated cytokines (excluding HSC medium supplements) was analyzed by ELISA within the supernatant cell culture medium. Data sets are normalized to internal positive controls and represent the average of 5 individual donors ± SD. (**B**) The amount of IL8 after 14 days in vitro on 2D PS, 3D PDMS and 3D SiOn was analyzed by ELISA within the supernatant cell culture medium. In addition, the IL8 secretion of cells grown on 2D PS within HSC cell culture medium without addition of valproic acid (VPA) and cytokines (SF), or just supplemented with cytokines (SF + Cytokines) or VPA (SF + VPA), was analyzed in comparison to the full HSC medium (full). Data sets are normalized to corresponding cell numbers (refer to Supplementary Fig. S8E,F) and represent the average of 6 individual donors ± SD. (**C**–**E**) The metabolism of cells after 14 days in vitro on 2D PS, 3D PDMS and 3D SiOn scaffolds was analyzed within Cell Mito Stress Tests using a Seahorse XFe96 Analyzer. (**C**) Oxygen consumption (OCR) and extracellular acidification rates (ECAR) were measured at basal conditions and after consecutive injection of oligomycin (ATP synthase inhibitor), FCCP (mitochondrial membrane uncoupling agent) and antimycin A (cytochrome *c* reductase inhibitor). (**D**) Bioenergetic parameters, i.e., the amount of OCR used for ATP production and the respiratory spare capacity were calculated from OCR data sets. (**E**) Energy phenotype maps were generated from OCR/ECAR data sets shown in (**C**). Basal and stressed (after oligomycin/FCCP injections) OCR/ECAR values are depicted for each group. Seahorse data sets were analyzed using Wave software and Microsoft Excel. (**B**–**E**) Represent the average of 3 individual donors ± SD. Statistical significances of data were tested using a two-tailed paired parametric t-test of 5 donors in (**A**) and 3 donors in (**D**,**E**). Statistics for this figure: *p < 0.05.

To validate the findings from transcriptomics and proteomics, we performed enzyme-linked immunosorbent assays (ELISAs), Seahorse and Western blot analysis. For cytokine measurements, we analyzed only those cytokines by ELISA that were not supplemented in the HSC medium and originated from cells during in vitro culture. We found that the pro-inflammatory cytokines IL1, IL17 and tumor necrosis factor (TNF)-α^30,31^ were only present in the medium of cells grown on 2D PS and 3D SiOn (Fig. 3A). Especially TNF-α, which is also associated with cell death and inflammation^31^, was highly abundant after 14 days in vitro on 3D SiOn. The pro-inflammatory IL6 and the anti-inflammatory IL10^30,31^ were detected only in the supernatant of cells from 2D PS and 3D PDMS. In addition, we observed that the pro-inflammatory interferon (IFN)-γ and granulocyte–macrophage colony-stimulating factor (GM-CSF), promoting the growth and differentiation of monocytes and dendritic cells^30,31^, were present in low amounts within the medium of cells on 2D PS and 3D PDMS scaffolds, but were highly enriched in the medium of 3D SiOn surfaces (Fig. 3A). Other cytokines like IL2, promoting immune cell activation and proliferation, or IL8, mediating the chemotaxis of immune cells^30,31^, were found in the supernatant of all cell culture scaffolds. Noteworthy, IL8 was in general the most abundant and significantly higher secreted cytokine in our ELISA-based analysis of cells after 14 days in vitro on 3D PDMS compared with 3D SiOn (*p* < 0.05) (Fig. 3A).

Thus, we studied the IL8 secretion during in vitro culture in more detail and investigated whether the HSC medium supplements, i.e. the panel of cytokines or VPA, influenced IL8 levels (Fig. 3B, Supplementary Fig. S8E,F). Only small amounts of IL8 were secreted from cells grown in HSC medium without cytokines/VPA (SF) or supplemented with VPA only (Fig. 3B). In contrast, cells grown in HSC medium supplemented just with cytokines showed the trend to secrete similar amounts of IL8 than cells grown in full HSC medium irrespective of the cell culture surface. Thus, the panel of cytokines within the HSC medium appeared to be mainly responsible for the high IL8 secretion. Our 3D PDMS scaffolds further promoted IL8 secretion during 14 days in vitro culture compared with 2D PS and 3D SiOn.

### Seahorse analysis shows a metabolic shift of cells expanded on 3D SiOn

Next, we performed metabolic flux analysis from HSCs expanded on 2D PS, 3D PDMS or 3D SiOn using Seahorse Analyzers (Fig. 3C–E). The Seahorse system enables to measure mitochondrial respiration (oxygen consumption rates; OCR) and glycolysis (extracellular acidification rates; ECAR) of cells in parallel and in real time. We observed that cells grown on 3D PDMS had lower basal OCR and similar ECAR values compared to 2D PS (Fig. 3C). Intriguingly, we found in cells expanded on 3D SiOn greatly decreased basal OCR but increased ECAR values compared to 2D PS and 3D PDMS. From Cell Mito Stress Tests, we calculated bioenergetic parameters, i.e., the amount of OCR used for ATP production and the respiratory spare capacity (Fig. 3D). Likewise, we found the ATP production in cells after 14 days in vitro on 3D SiOn greatly decreased (*p* < 0.05 to 3D PDMS), whereas the spare capacity was increased compared with the other two cell culture scaffolds. This indicates that 3D SiOn induces a metabolic shift from active respiration and mitochondrial ATP production into aerobic glycolysis compared to 2D PS or 3D PDMS (Fig. 3C,D). Analysis of the metabolic phenotype under basal and stressed conditions confirmed these observations (Fig. 3E).

### Western blot analysis confirms the activation of SREBP signaling in cells grown on 3D PDMS

**Figure 4:**
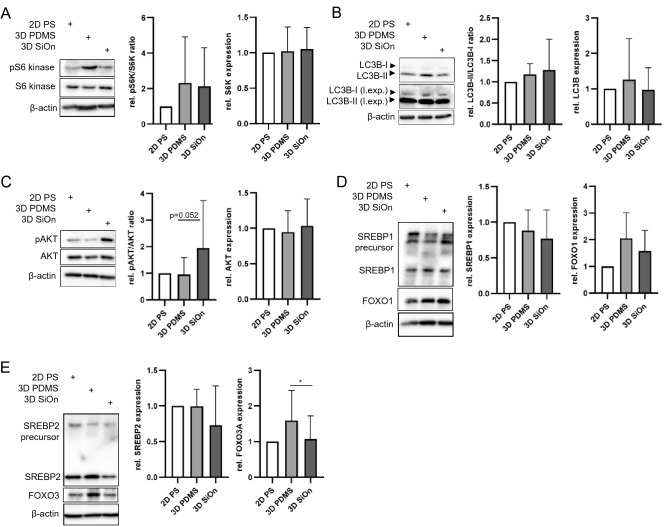
Human HSCs were cultivated for 14 days in vitro on 2D PS, 3D PDMS and 3D SiOn to expand high amounts of undifferentiated HSCs ex vivo. (**A**–**E**) After 14 DIV, the expression of indicated proteins was analyzed by Western blot. β-actin was used to control equal protein loading. (**A**,**C**) represent the average of 8 individual donors ± SD; (**B**) represents the average of 5 individual experiments ± SD; (**D**,**E**) represent the average of 4 individual donors ± SD. (l.exp.) indicates long exposures of immunoblots. Statistical significances of data were tested using a two-tailed paired parametric t-test of 8 donors in (**C**) and 4 donors in (**E**). Statistics for this figure: *p < 0.05.

Finally, we analyzed alterations of PI3K/AKT/mTOR pathways in cells after 14 days in vitro by Western blotting. mTOR, which is part in two functional complexes, namely mTORC1 and mTORC2 that share the same catalytic mTOR subunit but have unique regulatory subunits. mTORC1 comprises the regulatory-associated protein of mTOR (RAPTOR), and mTORC2 comprises the rapamycin-insensitive companion of mTOR (RICTOR)^32^. p70 (S6 kinase) is a direct target of mTORC1-mediated phosphorylation and reflects its activity^17,33,34^. We found that both 3D PDMS and 3D SiOn scaffolds slightly induced the phosphorylation of S6 kinase (pS6 kinase), whereas the total protein level of S6 kinase was not changed compared with 2D PS (Fig. 4A). In parallel, we observed a moderate increase of total LC3B level in cell grown on 3D PDMS and of the LC3B-II/LC3B-I ratio on 3D PDMS and 3D SiOn (Fig. 4B), which are both indicative for autophagy activation and reduced mTORC1 activity^33,34^. Interestingly, only cells expanded on 3D SiOn showed an increased phosphorylation of AKT at serine-437 (pAKT) (Fig. 4C), which is mediated by mTORC2^17^.

Finally, we investigated SREBP and FOXO signaling downstream of PI3K/AKT/mTOR pathways^21–23,27^ in cells after 14 days in vitro. The in vitro cultivation on 3D SiOn reduced the level of both SREBP1 and SREBP2 compared to 2D PS and 3D PDMS (Fig. 4D,E). In contrast, we saw that cells grown on 3D PDMS tended to have more active SREBP1/2^27,35^ (lower bands) and less of their precursor proteins (upper bands) compared with 2D PS and 3D SiOn (Fig. 4D,E). Furthermore, we observed that cells expanded on 3D PDMS had increased FOXO1 and strongly increased FOXO3 (*p* < 0.05 to 3D SiOn) protein levels compared to other cell culture scaffolds (Fig. 4D,E). These data indicate the activation of SREBP and FOXO signaling after 14 days in vitro on 3D PDMS scaffolds^21,23,27,35^, which correlates well with our multi-omics analysis in case of SREBP (Figs. 1A,B, 2, Supplementary Figs. S4, S6, S7).

In summary, our biochemical analysis together with our multi-omics approach indicate that 3D PDMS scaffolds are well suited to promote the growth of HSC via activation of SREBP, HIF1α and FOXO pathways, whereas SiOn-coated structures induce a pro-inflammatory response of the PI3K/AKT/mTOR signaling.

## Discussion

HSCT is the most effective treatment for a broad spectrum of hematopoietic malignancies as well as non-malignant congenital and acquired disorders. Since there is often a disparity between the number of HSCs needed by the patient and those available from the donor, an effective in vitro amplification prior to HSCT is required, but yet insufficiently realized^3,6^. For example, hematopoietic cell transplants from UCB are used worldwide to treat hematopoietic malignancies. UCB is rich in naïve HSC and CD34+ progenitor cells, but the total amount of cells in UCB is limited^4^. In this study, we confirm our previous findings^7^ that 3D PDMS scaffolds offer an optimal environment for human HSCs, which is characterized by a range of parameters, including an increased ability of HSC self-renewal and proliferation and to maintain their pluripotency. This is likely achieved by activating specific molecular pathways related to PI3K/AKT signaling, i.e., SREBP, HIF1α and FOXO pathways.

Human HSCs are semi-adherent, which is important for their homing and attachment in the BM compartment^36^ and HSC functions and behavior in vivo strongly depend on their microenvironment, the so-called stem cell niche^11–14^. Besides various interactions of HSCs with other cell types in the BM, the topological structures of the stem cell niche seems to be important for specific HSC differentiation and stem cell potential maintenance^11–14^. PS is currently the state-of-the-art material for HSC cultivation; however, the range of PS-based microscale 3D cell cultivation systems is limited. We directly compared 3D PDMS with 3D PS BM-like scaffolds and found that the 3D structure alone was insufficient to achieve high numbers of CD34+ cells and CD34+/CD38−/CD45RA−/CD49f+/CD90+ LT-HSCs. Only if grown on 3D PDMS, HSCs and the naïve sub-population of LT-HSCs were expanded efficiently. This confirms and extends the findings of our previous study^7^ showing that 3D PDMS scaffolds enable to amplify significantly higher numbers of CD34+ and LT-HSCs compared with other cell culture surfaces/scaffolds.

This effect between 3D PS and 3D PDMS might originate from their different biomechanical properties. The material stiffness strongly influences growth and differentiation of HSCs^37,38^. PS is a rigid/stiff material, which promotes the attachment, adhesion and migration of HSCs, which could facilitate the exit from the niche and thereby promotes the differentiation of HSCs during in vitro culture^37,38^.

Likewise, we showed previously that SiOn-coating of 3D PDMS scaffolds to reduce their hydrophobicity promotes the attachment of HSCs, but also their differentiation and reduces the numbers of LT-HSCs^7^. Here we show that this is accompanied by a pro-inflammatory immune response and activation of PI3K/AKT and mTOR (mTORC2) signaling pathways during 14 days in vitro culture. This correlates with the reduced clonogenic potential of cells expanded on 3D SiOn compared with 3D PDMS. Therefore, 3D SiOn scaffolds are not ideal for an in vitro HSC amplification before HSCT. In contrast, non-coated 3D PDMS is a soft substrate that is well suited to maintain HSC pluripotency during ex vivo expansion^37,39^. Cells grown on 3D PDMS showed the highest numbers of LT-HSCs, CFU-GEMM and BFU-E in both our current and previous study^7^. Noteworthy, BFU-E are primitive erythroid progenitors with high proliferative capacity and CFU-GEMM are multi-lineage progenitors, reflecting the immaturity of the cells^40^.

Thus, matrix elasticity and surface hydrophobicity of 3D cell culture systems are important factors and should be considered in attempts to propagate (LT)-HSCs in vitro for clinical applications. LT-HSCs are important for steady-state hematopoiesis up to several weeks after HSCT since this naïve subtype of HSCs has a highly conserved pluripotent character and capacity for self-renewal, whereas CD34+ HSCs and progenitors are important for a quick hematopoietic reconstitution after HSCT^29,41^.

In line with our previous study^7^, we found that our optimized HSC medium, particularly the panel of supplemented hematopoietic cytokines, is critical for cell expansion. During 14 days in vitro, cells secreted high amounts of IL8, and cells grown on 3D PDMS secreted highest level of IL8 compared with 2D PS and 3D SiOn. IL8 has been shown to activate HSC mobilization^42,43^. Therefore, it might be involved in the efficient cell expansion within our HSC medium. Nevertheless, the cell culture surfaces determined the efficiency of HSCs and LT-HSCs amplification, and 3D PMDS proofed to be the best within our setting. This was accompanied by a specific activation of molecular signaling pathways during in vitro cultivation. We observed the biggest alterations in the transcriptome and proteome of cells after 14 days in vitro compared to the CD34-enriched HSCs at day 0, which reflects the differentiation of a certain number of HSCs into other blood cell types in vitro. We found by IPA alterations regulating cell proliferation/cell cycle together with their upstream regulators, e.g., MYC^44,45^ and decreased signaling pathways, which are important for the maintenance and self-renewal of HSCs like NOTCH1 and FOXO1^21–23^ on all cell culture surfaces. Of note, 3D PDMS enabled to remain higher protein levels of FOXO1 and FOXO3 after 14 days in vitro compared to other surfaces. Additionally, IPA revealed that 3D PDMS and 3D SiOn scaffolds activated HIF1α signaling, another key molecular pathway for HSC maintenance^8,24–26^, but with strongest effect seen in cells grown on 3D PDMS.

FOXO and HIF1α pathways control the metabolism of HSCs^24,26,46,47^. Along this line, we found increased SREBP1/2 and FA/cholesterol metabolism by our multi-omics approach as well as moderately more of the active SREBP1/2 proteins by Western blot in cells grown on 3D PDMS after 14 days in vitro compared to day 0 and to other cell culture scaffolds. Our findings are consistent with the notion that an increased FA/cholesterol metabolism, mediated by SREBP and HIF1α signaling, is essential for the maintenance of HSC pluripotency during in vitro cultivation^8^. Moreover, growing cells require sufficient lipids for new membrane formation and expansion, and mTORC1 promotes the de novo lipid synthesis through SREBP transcription factors, which control the expression of metabolic genes involved in FA and cholesterol biosynthesis^48–50^.

Furthermore, this specific metabolic adaptation in cells grown on 3D PDMS is connected with a decreased mitochondrial respiration, which can be indicative of a higher number of immature HSCs^17,20,22–24^ and correlates to the increased amounts of LT-HSCs in cells expanded on 3D PDMS. Quiescent (or dormant) HSCs can be activated to enter the cell cycle and either self-renew or differentiate. Owing to their dormancy, the energy demand of quiescent HSCs is lower than that of proliferative progenitor cells, which might explain the lower respiration in cells expanded on 3D PDMS compared with 2D PS. In contrast, cells expanded on 3D SiOn had a greatly decreased respiration and increased glycolysis, which can be a sign for even higher numbers of quiescent, immature HSCs, but it is also a sign for inflammation and immune cell activation connected to the PI3K/AKT/mTOR signaling^20,22–24,26,46,47^. With respect to our previous data^7^ and the current study that showed reduced amounts of LT-HSCs and pro-inflammatory signaling in cells grown on 3D SiOn compared with 3D PDMS, we deduce that the shift into glycolysis was connected to inflammation.

Congruently, we detected the activation of an immune response to our cell culture conditions in cells after 14 days in vitro by IPA and ELISA. 3D PDMS leads to a balanced expression of pro- and anti-inflammatory cytokines, whereas 2D PS and especially 3D SiOn cell culture scaffolds induce the secretion of pro-inflammatory cytokines. Metabolic alterations during inflammation are mediated by PI3K/AKT pathways and the downstream mTOR/HIF1α signaling^26,46,47^, which is consistent to our findings.

mTOR is a master regulator of hematopoiesis^17,19,20^. IPA of our multi-omics analysis indicates that 14 days in vitro culture led to an activation of RICTOR, RAPTOR and mTORC1, but inactivation of TSC2-mediated molecular pathways, the latter most likely via AKT^19,32,33,51^. This indicated a general activation of mTOR, particularly mTORC1 in parallel to ongoing differentiation of HSCs in culture during their expansion, which is in line with the literature^17,19,20^. mTOR activation in general promotes lineage commitment and HSC depletion. Therefore, human HSCs are maintained ex vivo by GSK-3 and mTOR inhibition. In our case, we supplemented the HSC medium with VPA, which is known to inhibit GSK-3 and mTORC1 via Wnt activation^20,52^. Although mTORC1 hyper-activation contributes to HSC exhaustion and drives HSCs from quiescence into proliferation and differentiation, its activity is still required for HSC functions. Downstream effectors of mTORC1, notably S6 kinase, are important for HSC maintenance and function^17,18^. Therefore, a low basal activation of mTORC1 might be necessary to promote the amplification of HSCs. After 14 days in vitro we did not observe a clear trend for mTORC1 activation between the cell culture surfaces by IPA and Western blot. Nevertheless, we found an activation of mTORC2, judged by AKT phosphorylation specifically in cells grown on 3D SiOn compared with other cell culture scaffolds. Activation of AKT by phosphorylation occurs during inflammation^53,54^, which correlates to the pro-inflammatory status of these cells.

Taken together, cells grown on 3D PDMS activated FOXO, SREBP and HIF1α pathways, which increased their FA/cholesterol metabolism and reduced their mitochondrial respiration creating an optimal environment for in vitro HSC amplification. 3D SiOn though created a pro-inflammatory environment that activated PI3K/AKT, mTORC2 and HIF1α pathways inducing a metabolic shift into glycolysis. In consequence, only 3D PDMS enabled the growth and maintenance of high LT-HSC numbers and, thus, appears to be suitable for an ex vivo expansion of HSCs before HSCT. It remains to be elucidated which repopulating potential the HSCs amplified in this novel in vitro cultivation system offer in vivo. In addition, further studies should be done to optimize the biomechanical properties of 3D PDMS scaffolds and the cell culture conditions to further support the activation of HIF1α and SREBP signaling during ex vivo HSC expansion.

## Methods

### Cell culture

Cryoconserved pre-isolated human CD34+ HSCs were obtained from mobilized peripheral blood (mPB) of male and female healthy donors from the Cryogenic Storage Facility (Biobank) of the Children’s Hospital Jena. The study has been approved by the Jena University Hospital Ethics Committee (#3595-10/12, #5320-10/17).

Cells were cultured in HSC medium (Stemline^®^ II Hematopoietic Stem Cell Expansion Medium (Sigma Aldrich)) supplemented with 100 U/ml penicillin and 100 U/ml streptomycin (Lonza), 100 ng/ml SCF, 100 ng/ml TPO, 100 ng/ml FLT-3, 50 ng/ml IL-3 (all from Peprotech), 1 mM valproic acid (VPA; Sigma Aldrich) at 37 °C and 5% CO_2_ for an adaption period of 24 h prior to experiments (refer to Ref.^7^). For experiments, 1 × 10^4^ cells per well were seeded (day 0) into 24-well PS cell culture plates (Greiner) in 1 ml HSC medium and cultured for up to 14 days in vitro.

### Production of 3D PDMS and PS scaffolds

Three-dimensional (3D) polydimethylsiloxane (PDMS) structures were produced as described in Ref.^7^. Briefly, images of a microtome intersection from the bone marrow (BM) of a human long bone were analyzed, and silicon masters were fabricated and utilized as either casting or stamping molds. Production of the artificial scaffolds for HSC cultivation was done by casting and curing a thin silicone layer on top of the silicon BM masters. PDMS preparation was done following the manufacturer’s instructions (SYLGARD™ 184 Silicone Elastomer Kit, Dow Inc.). To achieve a more hydrophilic surface of the PDMS structure, a silicon oxide-based coating called Aquacer^®^ (APC GmbH; SiOn-covering) was used.

3D polystyrene (PS) structures were produced by hot embossing. A PDMS scaffold was coated with gold (2 µm) serving as a starting layer for galvanic refill and nickel was plated on top in an electroplating bath up to a thickness of 1 mm. The PDMS scaffold was afterwards removed with a special solvent (Dynasolve 218, Dynaloy Chemicals) in an ultrasonic bath. With the resulting nickel master, 3D PS BM-like structures were molded with a hot embossing machine (HEX02, Jenoptik Laser Optik Systeme GmbH). An embossing temperature of 125 °C and a demolding temperature of 65 °C was optimal.

### Cell cultivation on PDMS and PS structures

3D PDMS and 3D PS structures were washed in a descending series of ethanol dilutions (99%, 75%, 50% and 25% in H_2_O) and were placed into 24-well plates (Greiner). Cells were seeded into blank wells (2D PS) for control, SiOn-covered or uncovered 3D PDMS structures, as well as 3D PS structures. Cells were harvested for analysis by pipetting within the supernatant HSC medium.

### Flow cytometry of HSC surface markers

Analysis was done as described in Ref.^7^.

### Cell sorting by flow cytometry

Cells were harvested and washed with 1 ml PBS containing 2 mM EDTA and 5% HSA at 0 and 14 DIV. After washing in PBS containing 2 mM EDTA, 5% HSA and 1 µM Sytox blue (Thermo Scientific), viable Sytox-negative cells were sorted using a BD FACSAria Fusion with a 85 µm nozzle and FACSDiva 8.0.1 software (BD Bioscience). Filter set-up: 450/50 bandpass filter for Sytox. Gates were applied to exclude debris (Supplementary Fig. S9).

### Colony forming unit (CFU) assay

Assays were performed as described in Ref.^7^.

### Enzyme-linked immunosorbent assay (ELISA)

The Multi-Analyte ELISArray (Quiagen) and LEGEND MAX™ Human IL8 ELISA (BioLegend) kits were used following the manufacturer’s instructions.

### Preparation of protein lysates

1 × 10^5^ cells were resuspended per 10 µl of RIPA buffer [(50 mM Tris/HCl (pH 8.0), 150 mM NaCl, 1 mM EDTA, 1% Triton X-100, 1% sodium deoxycholate and 0.1% SDS] supplemented with PhosphoStop and protease inhibitor cocktail (PIC; both from Roche), and briefly sonicated (Bioruptor Plus, Diagenode) in 5 cycles (30 s ON/30 s OFF with high intensity at 4 °C).

### Immunoblotting

10–20 mg of protein per lane was separated by standard SDS–PAGE and transferred onto PVDF membranes. After blocking in blocking solution [100 mM Tris/HCl (pH 8.0), 450 mM NaCl, 5% dry milk and 0.05% Tween-20], the membranes were incubated with antibodies against: LC3B (1:2500), phospho-T389 p70 S6 kinase (1:2500), p70 S6 kinase (1:2500), phospho-S437 AKT (1:2500), FOXO3A (1:2500), FOXO1 (1:2500) (all from Cell Signaling), AKT (1:1000), SREBP-1 (1:1000) (both from Santa Cruz) and SREBP2 (1:1000; R&D Systems). Equal loading of protein was verified by the detection of β-Actin (1:10,000; Sigma Aldrich). Peroxidase-conjugated anti-mouse (1:5000), anti-rabbit (1:5000; both from KPL Sera Care) secondary antibodies were used for detection of specific signals with Pierce ECL Western Blotting Substrate (Thermo Scientific) (Supplementary Fig. S10).

### Seahorse analysis

7.5 × 10^4^ cells within 180 µl of Seahorse XF RPMI (Agilent Technologies; pH pre-adjusted to 7.4), supplemented with 10 mM d-glucose, 2 mM l-glutamine and 1 mM pyruvate (all from Thermo Scientific) were placed per well of a XF96 cell culture microplate (Agilent Technologies). Cells were incubated for 1 h in a CO_2_-free incubator at 37 °C and sedimented (5 min, 250×*g*, RT). Assays were performed on a Seahorse XFe96 Analyzer (Agilent Technologies). Basal oxygen consumption (OCR) and extracellular acidification rate (ECAR) were measured before adding 2 µM oligomycin (Sigma Aldrich), 2 µM carbonyl cyanide-*p*-trifluoromethoxy-phenylhydrazone (FCCP) (Abcam) and 2 µM antimycin A (Sigma Aldrich). Each compound was injected consecutively. OCR and ECAR were measured as pmoles/min and mpH/min respectively, in cycles of 3 min mix and 3 min measure periods at 37 °C. Wave software (Agilent Technologies) was used to analyze the datasets.

### Transcriptome analysis

#### Sample preparation and sequencing

RNA was isolated from HSCs using the RNeasy Mini Kit (Qiagen) following the manufacturer’s instructions. Sequencing of RNA samples was performed using Illumina’s next-generation sequencing methodology^55^. In brief, total RNA was quantified and quality checked using an Agilent 4200 Tapestation System in combination with RNA ScreenTape (both Agilent Technologies). Libraries were prepared from 300 ng of input material (total RNA) using NEBNext Ultra II Directional RNA Library Preparation Kit in combination with NEBNext rRNA Depletion Kit (Human/Mouse/Rat) and NEBNext Multiplex Oligos for Illumina (96 Unique Dual Index Primer Pairs) following the manufacturer's instructions (New England Biolabs). Quantification and quality check of libraries was done using an Agilent 4200 Tapestation System and D1000 ScreenTapes. Libraries were pooled and sequenced in one NovaSeq6000 SP 100 cycle run (101 cycle/single-end/standard loading workflow mode). Sequence information was converted to FASTQ format using bcl2fastq v2.20.0.422. Sequencing resulted in 62 × 10^6^ reads per sample on average.

#### Gene expression analysis

The RNA sequencing reads were aligned to the human reference genome (GRCh38 with the Ensembl genome annotation release 101) using STAR 2.7.6a (parameters: –alignIntronMax 100000, –outSJfilterReads Unique, –outSAMmultNmax 1, –outFilterMismatchNoverLmax 0.04)^56^. For each gene, all reads that map uniquely to one genomic position were counted with FeatureCounts 1.6.5 (multi-mapping or multi-overlapping reads were discarded, stranded mode was set to “–s 2”)^57^. Differentially expressed genes (DEGs) were determined with R 4.0.1 using the package DESeq2 1.28.1^58^. Only genes that have at least 1 read count in any of the analyzed samples of a particular comparison were subjected to DESeq2. HSCs cultured either on 2D PS, 3D PDMS or 3D SiOn for 14 days were compared to their initial state at day 0. In addition, the 14-days in vitro groups were compared with each other in a pairwise fashion: 3D PDMS versus 2D PS, 3D SiOn versus 2D PS and 3D PDMS versus 3D SiOn. In all 6 comparisons, the status “donor” was considered as a co-factor. For each gene in each comparison, the p-value was calculated using the Wald significance test. Resulting p-values were adjusted for multiple testing using the Benjamini & Hochberg correction. The log2 fold change (L2FC) values were shrunk with the DESeq2 function lfcShrink(type = "apeglm") to control for variance of L2FC estimates for genes with low read counts^59^. Genes with an adjusted *p* < 0.05 were considered differentially expressed.

### Proteome analysis

#### Sample preparation for proteomics analysis

For proteomics analysis, samples were sonicated (Bioruptor Plus, Diagenode) for 10 cycles (30 s ON/60 s OFF) at high intensity, at 20 °C, followed by boiling at 95 °C for 5 min. Reduction was followed by alkylation with iodoacetamide (IAA, final concentration 15 mM) for 30 min at RT in the dark. Protein amounts were estimated following an SDS-PAGE gel of 10 µl of each sample against an in-house cell lysate of known quantity. 30 µg of each sample was taken along for digestion. Proteins were precipitated overnight at − 20 °C after addition of 8 × volume of ice-cold acetone. The following day, the samples were centrifuged at 20,800×*g* for 30 min at 4 °C and the supernatant carefully removed. Pellets were washed twice with 300 µl ice-cold 80% (v/v) acetone in water, then centrifuged at 20,800×*g* at 4 °C for 10 min. After removing the acetone, pellet were air-dried before addition of 25 µl of digestion buffer (1 M Guanidine, 100 mM HEPES, pH 8). Samples were resuspended with sonication as explained above, then LysC (Wako) was added at 1:100 (w/w) enzyme:protein ratio and digestion proceeded for 4 h at 37 °C under shaking (1000 rpm for 1 h, then 650 rpm). Samples were then diluted 1:1 with MilliQ water and trypsin (Promega) added at 1:100 (w/w) enzyme:protein ratio. Samples were further digested overnight at 37 °C under shaking (650 rpm). The day after, digests were acidified by the addition of TFA to a final concentration of 10% (v/v), heated at 37 °C and then desalted with Waters Oasis^®^ HLB µElution Plate 30 µm (Waters Corporation, MA, USA) under a soft vacuum following the manufacturer's instruction. Briefly, the columns were conditioned with 3 × 100 µl solvent B (80% (v/v) acetonitrile; 0.05% (v/v) formic acid) and equilibrated with 3 × 100 µl solvent A (0.05% (v/v) formic acid in Milli-Q water). The samples were loaded, washed 3 times with 100 µl solvent A, and then eluted into 0.2 ml PCR tubes with 50 µl solvent B. The eluates were dried down using a speed vacuum centrifuge (Eppendorf Concentrator Plus, Eppendorf AG). Dried samples were stored at − 20 °C until analysis.

#### LC–MS analysis

Prior to analysis, samples were reconstituted in MS Buffer (5% acetonitrile, 95% Milli-Q water, with 0.1% formic acid) and spiked with iRT peptides (Biognosys). Peptides were separated in trap/elute mode using the nanoAcquity MClass Ultra-High Performance Liquid Chromatography system (Corporation) equipped with a trapping (nanoAcquity Symmetry C18, 5 μm, 180 μm × 20 mm) and an analytical column (nanoAcquity BEH C18, 1.7 μm, 75 μm × 250 mm). Solvent A was water and 0.1% formic acid, and solvent B was acetonitrile and 0.1% formic acid. 1 µl of the sample (∼ 1 μg on column) were loaded with a constant flow of solvent A at 5 μl/min onto the trapping column. Trapping time was 6 min. Peptides were eluted via the analytical column with a constant flow of 0.3 μl/min. During the elution, the percentage of solvent B increased in a nonlinear fashion from 0 to 40% in 120 min. Total run time was 145 min including equilibration and conditioning. The LC was coupled either to an Orbitrap Q-Exactive HFX or to an Orbitrap Exploris 480 (Thermo Scientific) using the Proxeon nanospray source. The peptides were introduced into the mass spectrometer via a Pico-Tip Emitter 360-μm outer diameter × 20-μm inner diameter, 10-μm tip (New Objective) heated at 300 °C, and a spray voltage of 2.2 kV was applied. The capillary temperature was set at 300 °C. The radio frequency ion funnel was set to 30%.

For sample-specific spectral library generation, pooled samples of the different condition were analyzed additionally by data-dependent acquisition (DDA). Full scan MS spectra with mass range 375–1650 *m/z* (using quadrupole isolation) were acquired with resolution of 60,000 FWHM. The filling time was set at maximum of 20 ms with limitation of 3 × 10^6^ ions. The “Top Scan” method was employed to take the maximum number of precursor ions (with an intensity threshold of 4.4 × 104) from the full scan MS for fragmentation (using HCD collision energy, 27%) and quadrupole isolation (1.4 Da window) with a cycle time of 20 scans. The MIPS (monoisotopic precursor selection) peptide algorithm was employed. MS/MS data were acquired with a resolution of 15,000 FWHM and a fixed first mass of 120 *m/z*. The filling time was set at maximum of 25 ms with limitation of 2 × 10^5^ ions. Only multiply charged (2+ − 7+) precursor ions were selected for MS/MS. Dynamic exclusion was employed with maximum retention period of 15 s and relative mass window of 10 ppm. Isotopes were excluded.

For data independent acquisition (DIA), full scan mass spectrometry (MS) spectra with mass range 350–1650 *m/z* were acquired in profile mode in the Orbitrap with resolution of 120,000 FWHM. The default charge state was set to 3+. The filling time was set at maximum of 60 ms with limitation of 3 × 10^6^ ions. DIA scans were acquired with 40 mass window segments of differing widths across the MS1 mass range. Higher collisional dissociation fragmentation (stepped normalized collision energy; 25, 27.5, and 30%) was applied, and MS/MS spectra were acquired with a resolution of 30,000 FWHM with a fixed first mass of 200 *m/z* after accumulation of 3 × 10^6^ ions or after filling time of 35 ms (whichever occurred first). All data were acquired in profile mode. For data acquisition and processing of the raw data, Xcalibur 4.0 (Thermo Scientific) and Tune version 2.9 were used.

#### Data processing

Acquired data were processed using Spectronaut Professional v13.10 (Biognosys AG). For library creation, the DDA and DIA raw files were searched with Pulsar (Biognosys AG) against the human UniProt database (Homo sapiens, 20,186 entries, release 2016_01) with a list of common contaminants appended, using default settings. For library generation, default BGS factory settings were used.

DIA data were searched against this spectral library using BGS factory settings, except: Proteotypicity Filter = Only Protein Group Specific; Major Group Quantity = Median peptide quantity; Major Group Top N = OFF; Minor Group Quantity = Median precursor quantity; Minor Group Top N = OFF; Data Filtering = Qvalue sparse; Normalization Strategy = Local normalization; Row Selection = Automatic. The same parameters were used for data run in directDIA only.

The candidates and protein report tables were exported from Spectronaut and used for volcano plots generation and Principal Component Analysis (PCA), respectively, using R version 3.4.1 and RStudio server version 1.1.463. Protein groups were considered as significantly enriched in if they displayed a Q value < 0.01 and average log2 ratio > 1.5.

### Molecular pathway analysis

The data sets from transcriptome and proteome analyses were post-analyzed using Ingenuity Pathway Analysis (IPA) software (Qiagen). Heat maps of ingenuity canonical pathways and upstream regulators were created using Excel.

### Statistical analysis

Results are displayed as mean values ± standard deviation (SD) using GraphPad Prism (GraphPad Software). Statistical significances of data were tested using a two-tailed paired parametric t-test using GraphPad Prism if not mentioned otherwise (*p < 0.05).

## Supplementary Information

Supplementary Information.

## Data Availability

The mass spectrometry proteomics data sets have been deposited to the ProteomeXchange Consortium (http://proteomecentral.proteomexchange.org) via the PRIDE partner repository^60,61^ with the data set identifiers PXD025849. Read counts of the RNA-seq data generated and discussed in this study as well as derived DESeq2 results have been deposited in NCBI's Gene Expression Omnibus^62^ and are accessible through GEO Series accession number GSE178657 (https://www.ncbi.nlm.nih.gov/geo/query/acc.cgi?acc=GSE178657). Otherwise, all data generated or analyzed during this study are included in this published article (and its Supplementary Information files).
