# Supplementary material for: Molecular characterization of hematopoietic stem cells after in vitro amplification on biomimetic 3D PDMS cell culture scaffolds
Source: Sci Rep. 2021 Oct 27;11:21163. doi: 10.1038/s41598-021-00619-6 (PMC8551314; doi:10.1038/s41598-021-00619-6)
Supplement: Supplementary file 1 — Supplementary Information. [file 41598_2021_619_MOESM1_ESM.pdf]

Supplementary figures 1-10
Marx-Blümel *et al*., 2021

Supplementary Figure 1

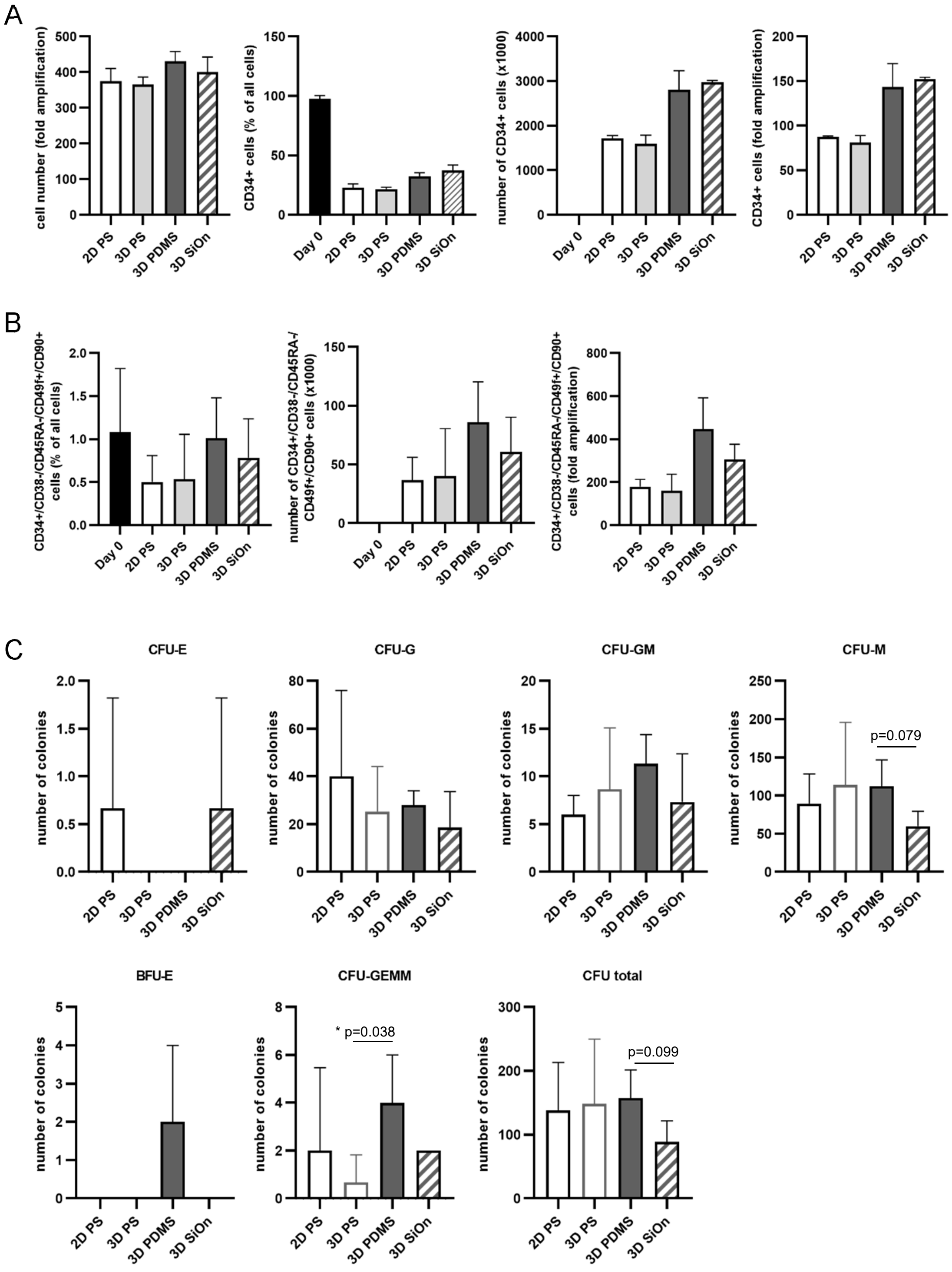

See next page for caption.

Figure S1

Human HSCs were cultivated for 14 days *in vitro* on 2D PS, 3D PS, 3D PDMS and 3D SiOn to expand high amounts of undifferentiated HSCs *ex vivo*. CD34+ and CD34+/ CD38-/ CD45RA-/ CD49f+/ CD90+ cells were determined by flow cytometric analyses using a combination of specific antibodies, and vital cells were selected using DAPI. CFUs were counted after additional 14 days of incubation in multi-lineage CFU medium. (A) The amplification of all cells and CD34+ cells, the percentage and absolute number of CD34+ cells are depicted. (B) The amplification, percentage and the absolute cell number of CD34+/ CD38-/ CD45RA-/ CD49f+/ CD90+ cells are depicted. (C) Total numbers of CFU, colony-forming unit-granulocyte/erythroid/megakaryocyte/monocyte (CFU-GEMM), colony-forming unit-granulocyte/ macrophage (CFU-GM), burst-forming unit-erythroid (BFU-E), and colony-forming unit-erythroid (CFU-E), colony-forming unit-macrophage (CFU-M), colony-forming unit-granulocyte (CFU-G) per $1x10^4$ seeded human HSCs are depicted. Thereby, CFU-E are clonogenic progenitors containing clusters of hemoglobinized erythroblasts presenting more mature erythroid progenitors with less proliferative capacity. In contrast, BFU-E are primitive erythroid progenitors with high proliferative capacity. CFU-G are clonogenic progenitors of granulocytes that give rise to a homogeneous population of eosinophils, basophils or neutrophils, whereas CFU-M are clonogenic progenitors of macrophages that give rise to a homogenous population of macrophages. CFU-GM are progenitors that give rise to colonies containing a heterogeneous population of macrophages and granulocytes with a morphology similar to CFU-M and CFU-G. CFU-GEMM are multi-lineage progenitors that give rise to erythroid, granulocyte, macrophage and megakaryocyte lineages, reflecting the immaturity of the cells. (A-B) represent the average of 2 individual donors ± SD. (C) represents the average of 3 individual donors ± SD. Statistical significances of data were tested using a two-tailed paired parametric t-test of 3 donors in (C). Statistics for this figure: * p <0.05.

Supplementary Figure 2

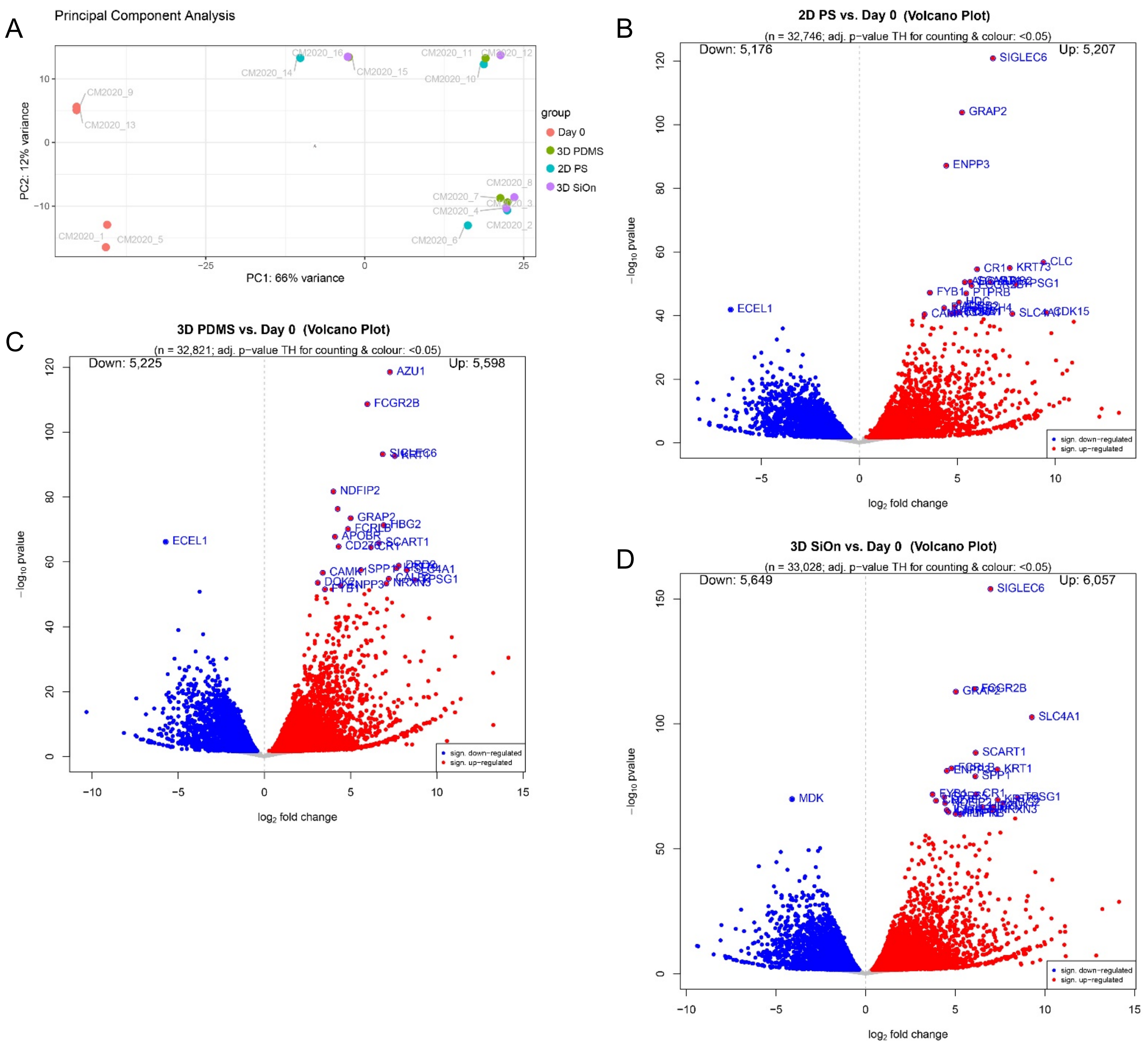

Figure S2

Human HSCs were cultivated for 14 days *in vitro* on 2D PS, 3D PDMS and 3D SiOn to expand high numbers of undifferentiated HSCs ex vivo. The transcriptome of the initial HSC population at day 0 and after 14 days *in vitro* on 2D PS, 3D PDMS and 3D SiOn scaffolds was analyzed by RNA sequencing (with 3 replicates per group). (A) A principal component analysis (PCA) was done on the transcriptome data set. The three individual replicates of each group are shown: Day 0 in magenta, 3D PDMS in green, 2D PS in blue and 3D SiOn in purple. (B-C) Volcano plots show highly altered transcripts (cutoff: adjusted $p < 0.05$) after 14 days *in vitro* cell culture on 2D PS (B), 3D PDMS (C) and 3D SiOn (D) compared to day 0 (blue decrease, red increase, grey not significant, only the 25 most significant genes are highlighted and labelled with gene symbols if one exists).

Supplementary Figure 3

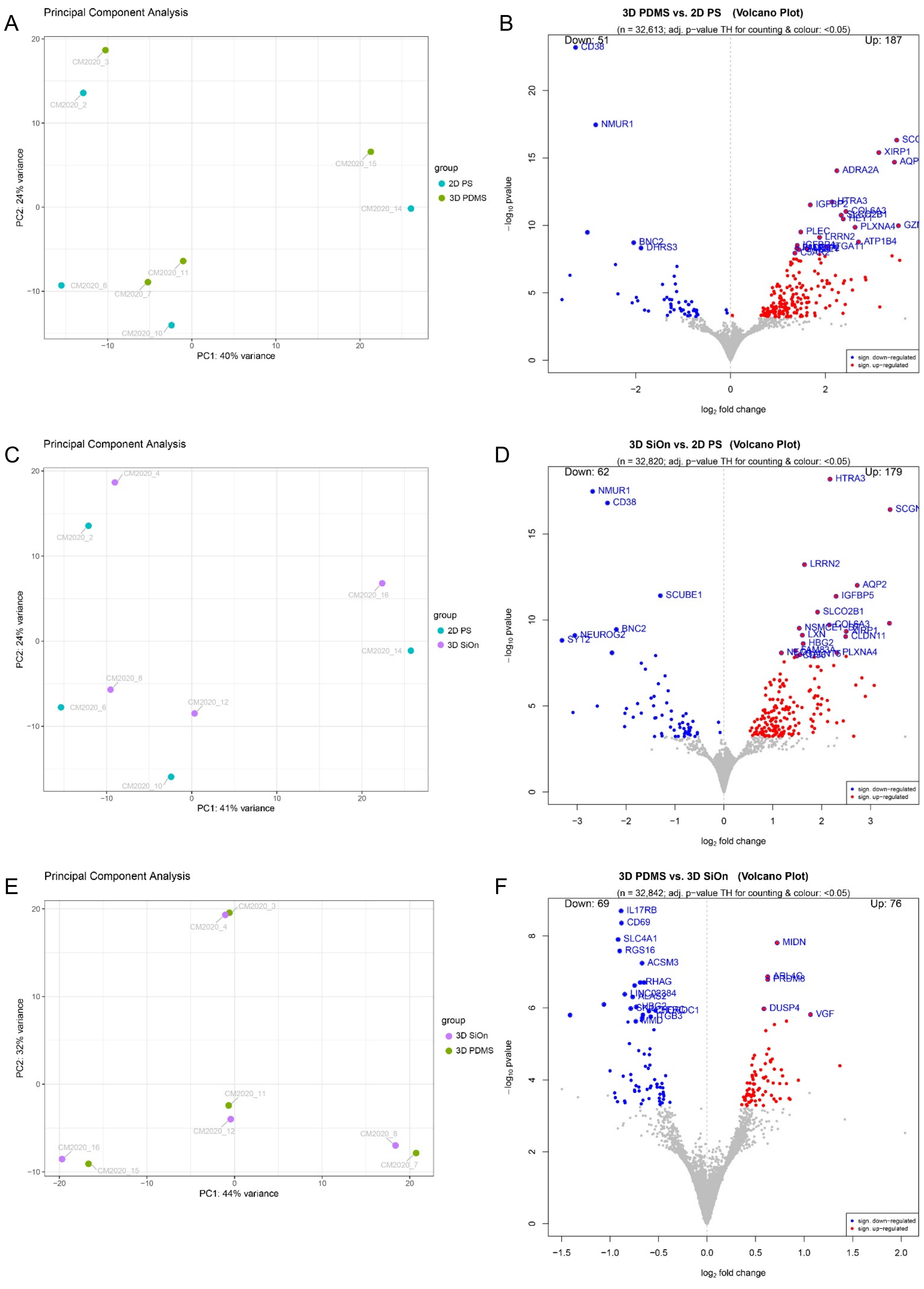

See next page for caption.

Figure S3

Human HSCs were cultivated for 14 days *in vitro* on 2D PS, 3D PDMS and 3D SiOn to expand high numbers of undifferentiated HSCs ex vivo. The transcriptome of the initial HSC population at day 0 and after 14 days *in vitro* on 2D PS, 3D PDMS and 3D SiOn scaffolds was analyzed by RNA sequencing (with 3 replicates per group). (A, C & E) Principal component analyses (PCA) were done on the transcriptome data set for the comparisons of 2D PS with 3D PDMS (A), 2D PS with 3D SiOn (C) and 3D PDMS with 3D SiOn (E). The three individual replicates of each group are shown: 3D PDMS in green, 2D PS in blue and 3D SiOn in purple. (B, D & F) Volcano plots show highly altered transcripts (cutoff: adjusted $p < 0.05$) for the comparisons 3D PDMS vs. 2D PS (B), 3D SiOn vs. 2D PS (D) and 3D PDMS vs. 3D SiOn (F) (blue decrease, red increase, grey not significant, only the 25 most significant genes are highlighted and labelled with gene symbols if one exists).

Supplementary Figure 4

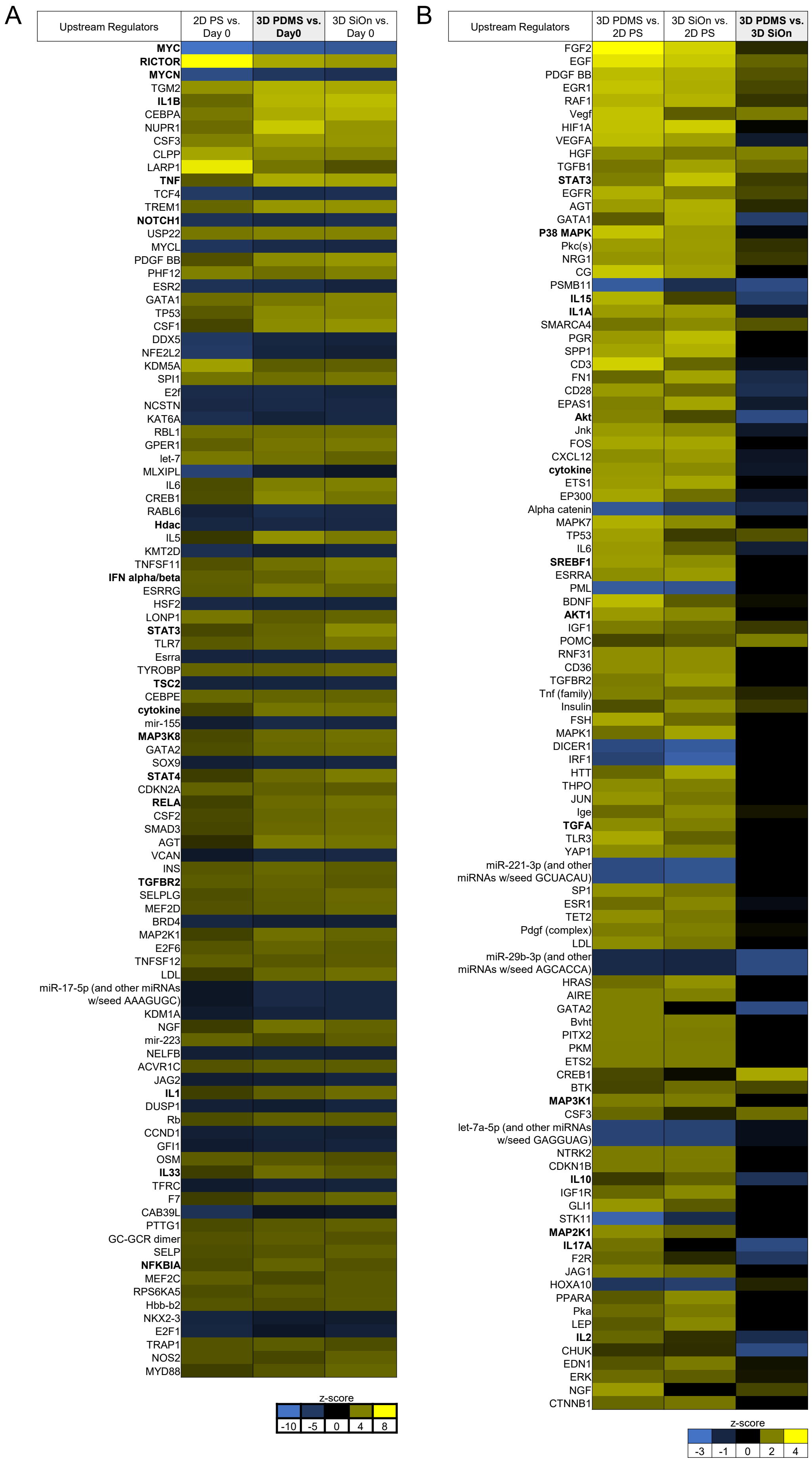

See next page for caption.

Figure S4

Human HSCs were cultivated for 14 days *in vitro* on 2D PS, 3D PDMS and 3D SiOn to expand high numbers of undifferentiated HSCs *ex vivo*. The transcriptome of the initial HSC population at day 0 and after 14 days *in vitro* on 2D PS, 3D PDMS and 3D SiOn scaffolds was analyzed by RNA sequencing (with 3 replicates per group) and the data sets were post-analyzed using IPA. (A) Activation of regulatory molecules (upstream regulators; top 100) after 14 days *in vitro* on 2D PS, 3D PDMS and 3D SiOn scaffolds compared to day 0 (analysis cutoff: $q < 0.05$, log2-fold change >1) and (B) activation of regulatory molecules (upstream regulators; top 100) after 14 days *in vitro* in comparison between 2D PS, 3D PDMS and 3D SiOn scaffolds (analysis cutoff: $q < 0.05$) is indicated by corresponding z-scores (blue inactivation/yellow activation) and ranked by the -log(p-value) of individual molecule (most significant at the top). Important pathways/molecules are highlighted with bold text.

Supplementary Figure 5

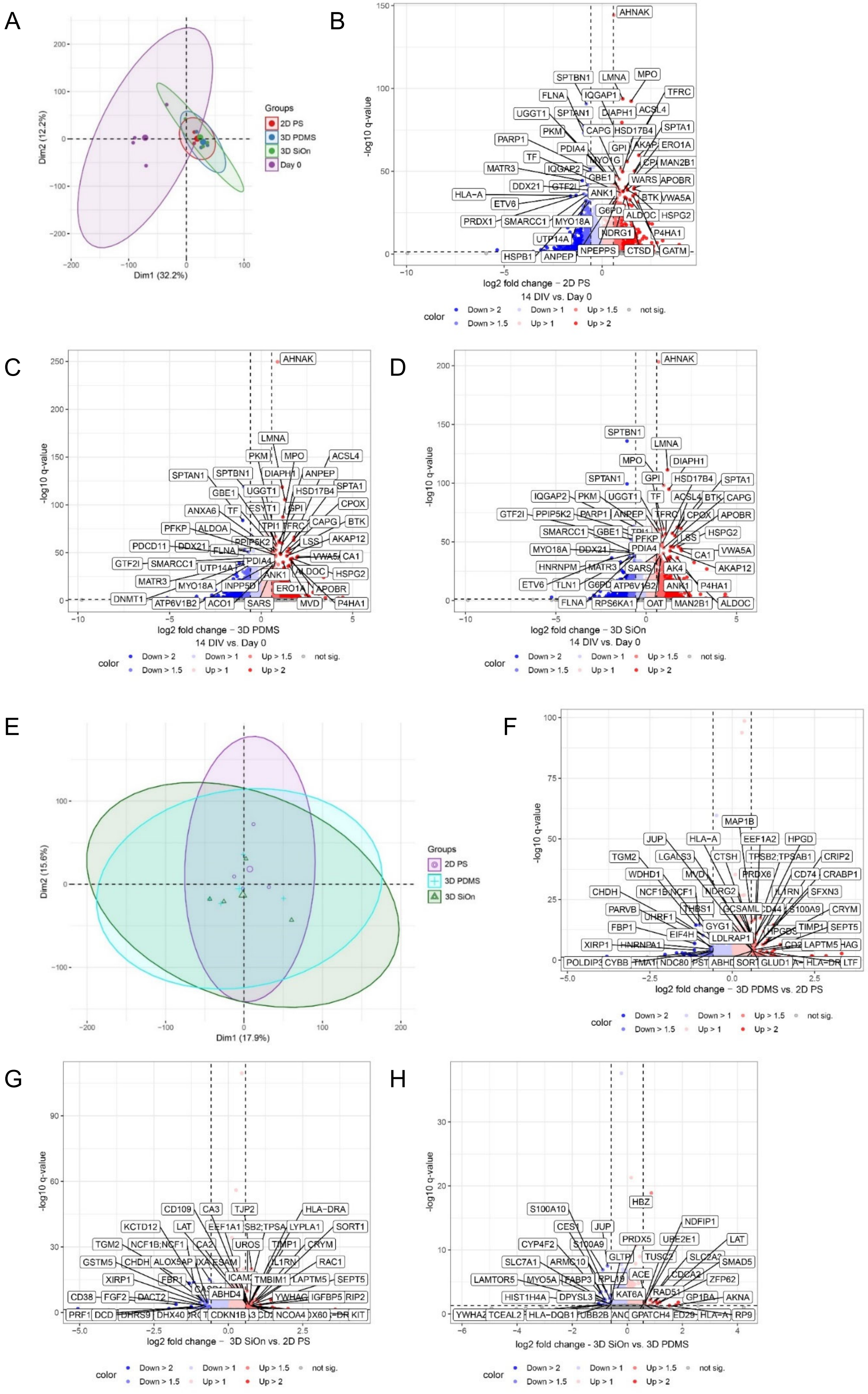

See next page for caption.

Figure S5

Human HSCs were cultivated for 14 days *in vitro* on 2D PS, 3D PDMS and 3D SiOn to expand high numbers of undifferentiated HSCs *ex vivo*. The proteome of the initial HSC population at day 0 and after 14 days *in vitro* on 2D PS, 3D PDMS and 3D SiOn scaffolds was analyzed by untargeted mass spectrometry (with 4 replicates per group). (A) A principal component analysis (PCA) was done on the proteome data set using R studio. The four individual replicates of each group are shown together with their median value (biggest symbol): 2D PS in red, 3D PDMS in blue, 3D SiOn in green and Day 0 in purple. (B-C) Volcano plots show highly altered proteins (cutoff: $q < 0.05$; log2-fold change >0.5) after 14 days *in vitro* cell culture on 2D PS (B), 3D PDMS (C) and 3D SiOn (D) compared to day 0 (blue decrease/red increase). (E) From the samples at 14 days *in vitro*, a second principal component analysis (PCA) was done on the proteome data set using R studio: 2D PS in purple, 3D PDMS in turquois and 3D SiOn in green. (F-H) Volcano plots show highly altered proteins (cutoff: $q < 0.05$; log2-fold change >0.5) for the comparisons between 3D PDMS vs. 2D PS (F), 3D SiOn vs. 2D PS (G) and 3D SiOn vs. 3D PDMS (H) (blue decrease/red increase).

Supplementary Figure 6

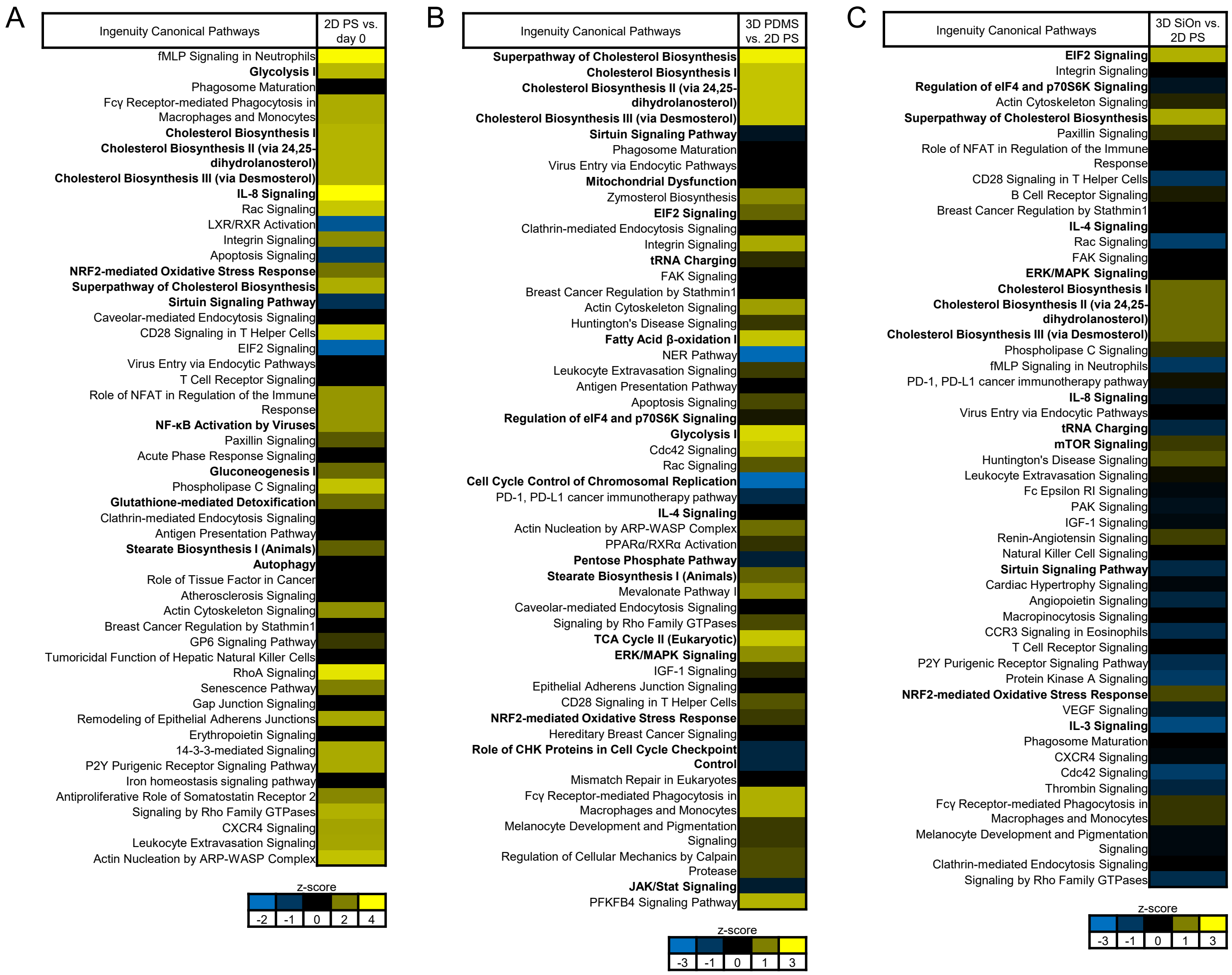

Figure S6

Human HSCs were cultivated for 14 days *in vitro* on 2D PS, 3D PDMS and 3D SiOn to expand high numbers of undifferentiated HSCs *ex vivo*. The proteome of the initial HSC population at day 0 and after 14 days *in vitro* on 2D PS, 3D PDMS and 3D SiOn scaffolds was analyzed by untargeted mass spectrometry (with 4 replicates per group) and the data sets were post-analyzed using IPA. (A) Activation of cellular pathways (top 50) after 14 days *in vitro* on 2D PS surfaces compared to day 0 (analysis cutoff: $q < 0.05$, log2-fold change >0.5), and activation of cellular pathways (top 50) after 14 days *in vitro* in comparison of 3D PDMS vs. 2D PS (B) and 3D SiOn vs. 2D PS (C) scaffolds (analysis cutoff: $q < 0.05$) is indicated by corresponding z-scores (blue inactivation/yellow activation) and ranked by the -log(p-value) of individual pathways (most significant at the top). Important pathways/molecules are highlighted with bold text.

Supplementary Figure 7

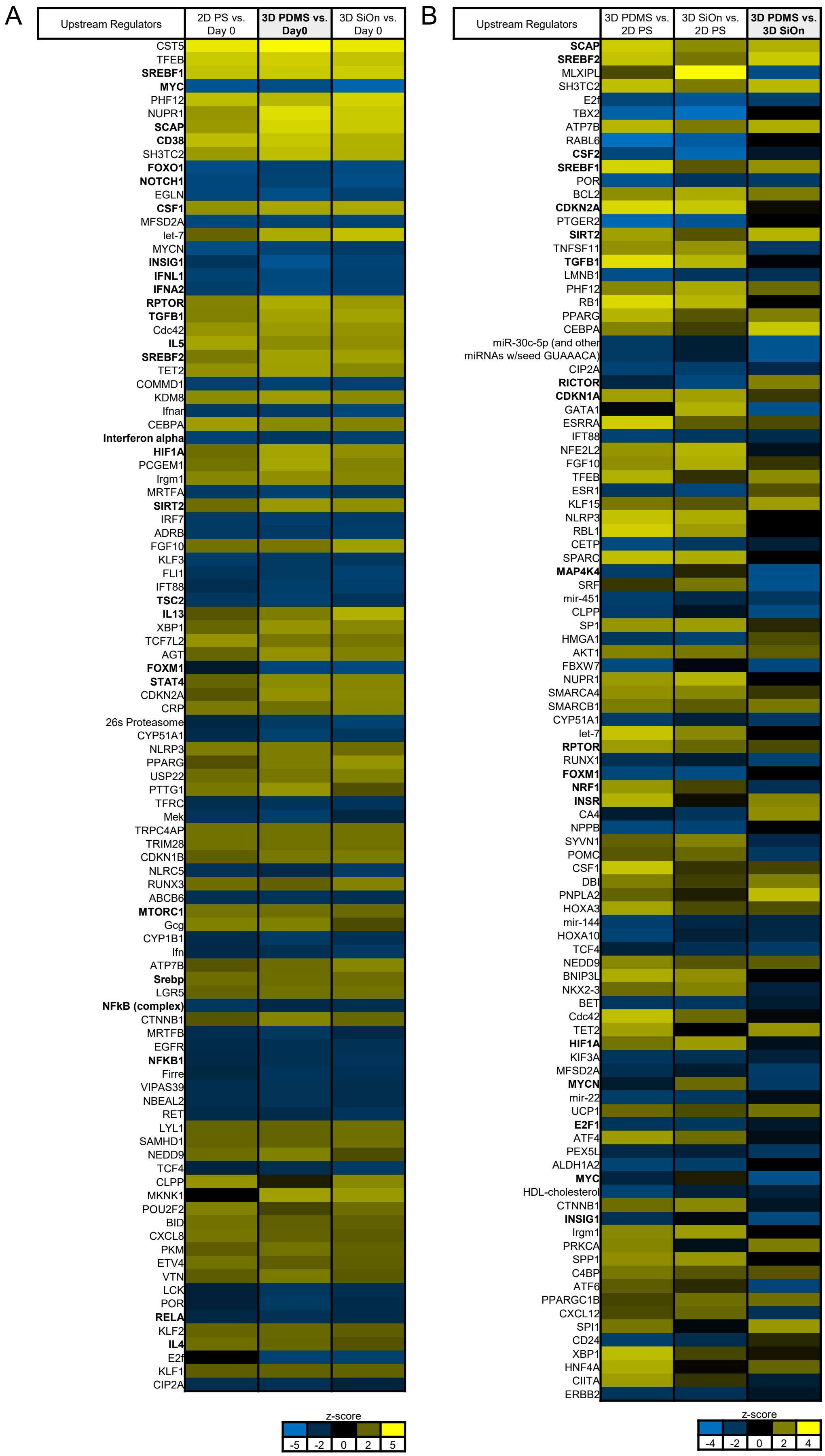

See next page for caption.

Figure S7

Human HSCs were cultivated for 14 days *in vitro* on 2D PS, 3D PDMS and 3D SiOn to expand high numbers of undifferentiated HSCs *ex vivo*. The proteome of the initial HSC population at day 0 and after 14 days *in vitro* on 2D PS, 3D PDMS and 3D SiOn scaffolds was analyzed by untargeted mass spectrometry (with 4 replicates per group) and the data sets were post-analyzed using IPA. (A) Activation of regulatory molecules (upstream regulators; top 100) after 14 days *in vitro* on 2D PS, 3D PDMS and 3D SiOn scaffolds compared to day 0 (analysis cutoff: $q < 0.05$, log2-fold change >0.5) and (B) activation of regulatory molecules (upstream regulators; top 100) after 14 days *in vitro* in comparison between 2D PS, 3D PDMS and 3D SiOn scaffolds (analysis cutoff: $q < 0.05$) is indicated by corresponding z-scores (blue inactivation/yellow activation) and ranked by the -log(p-value) of individual molecule (most significant at the top). Important pathways/molecules are highlighted with bold text.

Supplementary Figure 8

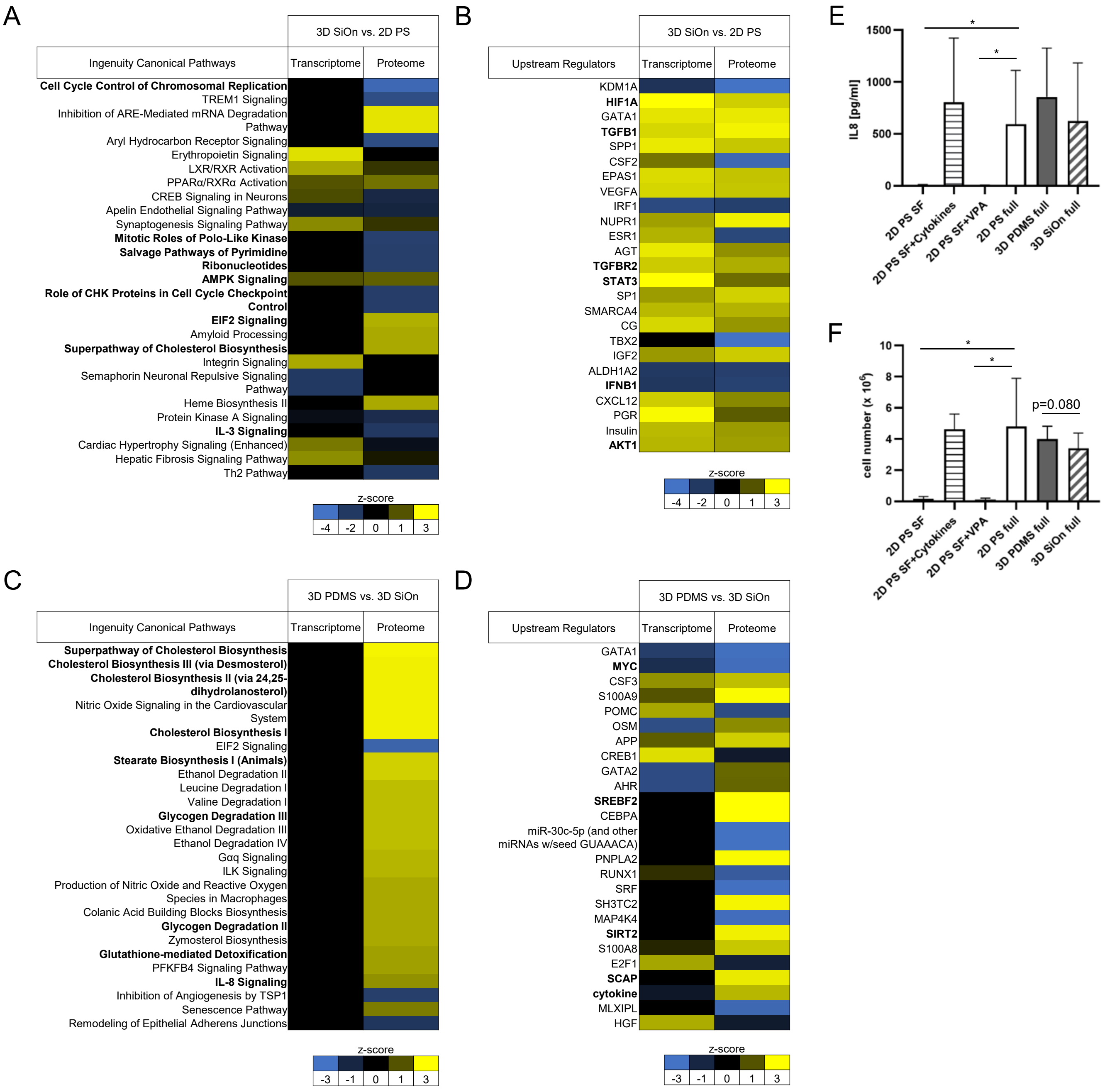

Figure S8

Human HSCs were cultivated for 14 days *in vitro* on 2D PS, 3D PDMS and 3D SiOn to expand high numbers of undifferentiated HSCs *ex vivo*. The transcriptome and proteome of the initial HSC population at day 0 and after 14 days *in vitro* on 2D PS, 3D PDMS and 3D SiOn scaffolds was analyzed by RNA sequencing (with 3 replicates per group) and untargeted mass spectrometry (with 4 replicates per group), respectively, and the data sets were post-analyzed using IPA. (A-D) Transcriptome and proteome data sets were compared using IPA (analysis cutoff: q <0.05). (A) Activation of similar cellular pathways (top 25) and (B) activation of similar regulatory molecules (upstream regulators; top 25) after 14 days *in vitro* in comparison of 3D SiOn vs. 2D PS scaffolds are indicated by corresponding z-scores and ranked by the -log(p-value) of individual molecules. (C) Activation of similar cellular pathways (top 25) and (C) activation of similar regulatory molecules (upstream regulators; top 25) after 14 days *in vitro* in comparison of 3D PDMS vs. 3D SiOn scaffolds are indicated by corresponding z-scores and ranked by the -log(p-value) of individual molecules. Important pathways/molecules are highlighted with bold text. (E) The concentration of IL8 after 14 days *in vitro* on 2D PS, 3D PDMS and 3D SiOn was analyzed by ELISA within the supernatant cell culture medium. (F) In parallel, cell numbers were counted within corresponding cell culture wells for normalization of IL8 levels (refer to Fig. 3B). (E-F) In addition, the concentration of IL8 in the supernatant and number of cells grown on 2D PS within HSC cell culture medium without addition of valproic acid (VPA) and cytokines (SF), or just supplemented with cytokines (SF+Cytokines) or VPA (SF+VPA), was analyzed in comparison to the full HSC medium (full). (E-F) represent the average of 6 individual donors ± SD. Statistical significances of data were tested using a two-tailed paired parametric t-test of 6 donors in (E-F). Statistics for this figure: * p <0.05.

Supplementary Figure 9

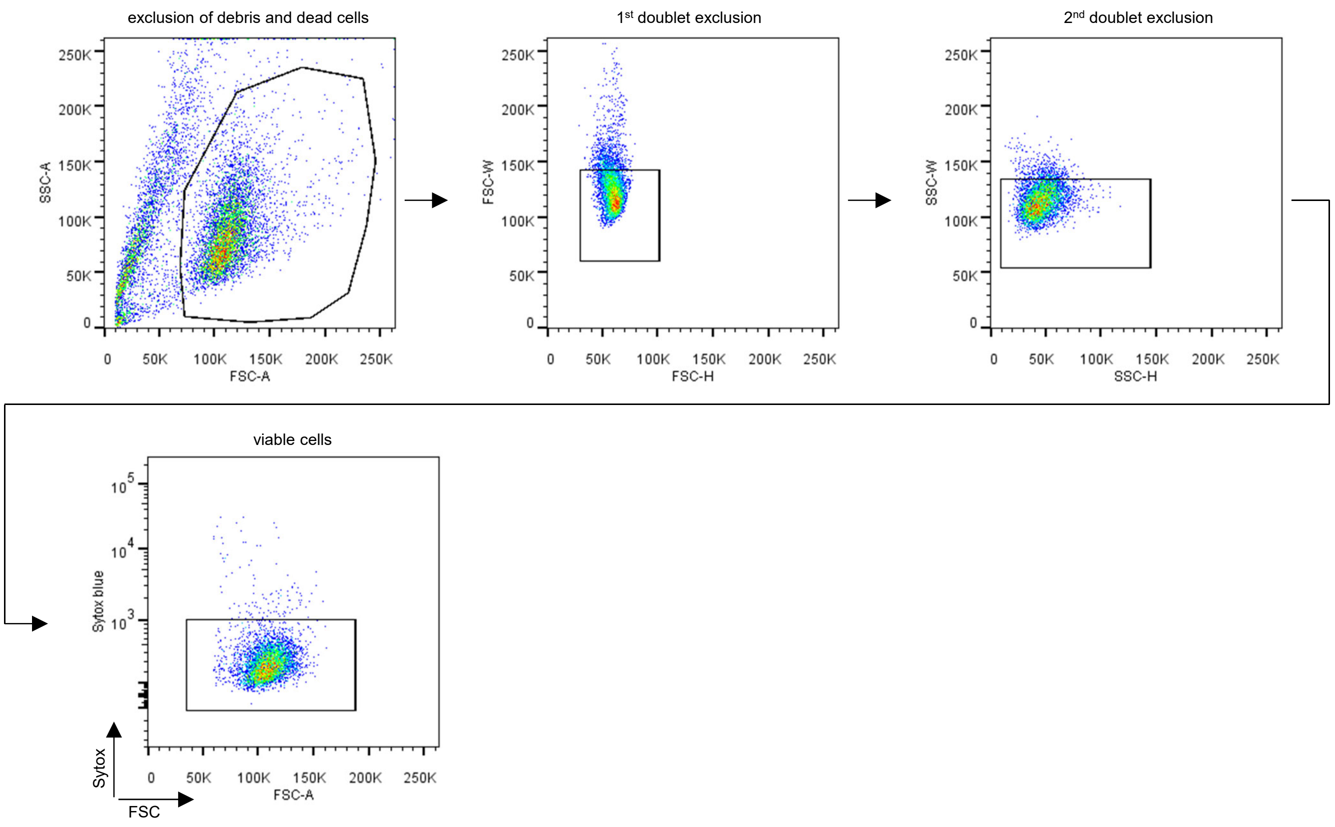

Figure S9
Cell sorting after 14 days *in vitro* by flow cytometry. Gates were applied and viable Sytox-negative cells were sorted to exclude cell debris, dead or doublet cells using a BD FACSAria Fusion and FACSDiva 8.0.1 software.

Supplementary Figure 10

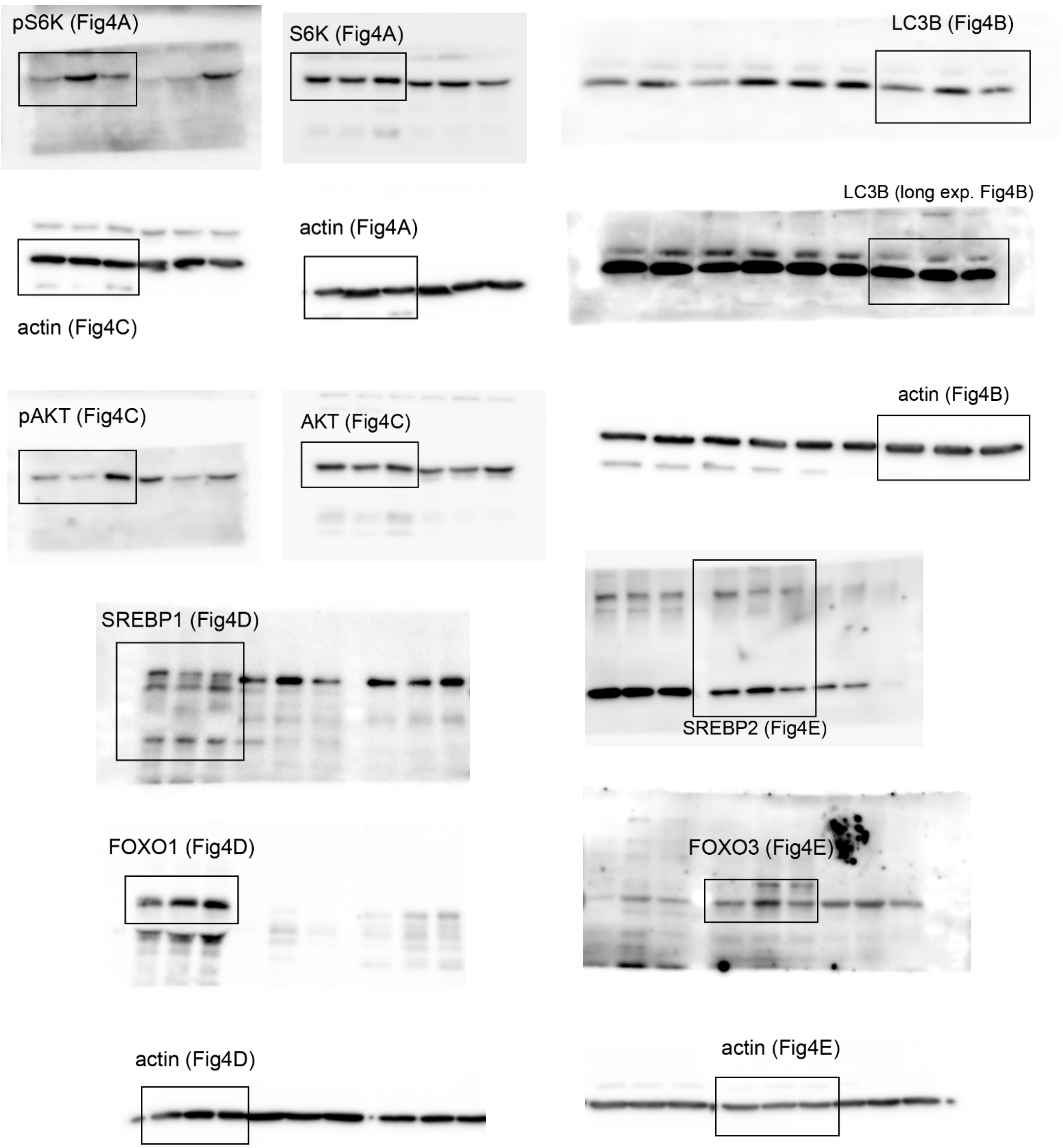

Figure S10
Western blot raw data of figures 4A-E.
